# Immune-Related Molecular Profiling of Thymoma With Myasthenia Gravis

**DOI:** 10.3389/fgene.2021.756493

**Published:** 2021-10-28

**Authors:** Jinman Zhuang, Maohao Guan, Maolin Liu, Yuhang Liu, Shuyan Yang, Zhijian Hu, Fancai Lai, Fei He

**Affiliations:** ^1^ Department of Epidemiology and Health Statistics, School of Public Health, Fujian Medical University, Fuzhou, China; ^2^ Fujian Provincial Key Laboratory of Tumor Microbiology, Fujian Medical University, Fuzhou, China; ^3^ Fujian Digital Tumor Data Research Center, Fuzhou, China; ^4^ Department of Thoracic Surgery, The First Affiliated Hospital of Fujian Medical University, Fuzhou, China

**Keywords:** thymoma, mg, bioinformatics, immune-related lncRNAs, functional enrichment analysis

## Abstract

**Background:** Approximately 50% of thymoma patients also show myasthenia gravis (MG), which is an autoimmune disease; however, the pathogenesis of MG-associated thymoma remains elusive. Our aim was to investigate immune-related lncRNA profiles of a set of candidate genes for better understanding of the molecular mechanism underlying the pathogenesis of thymoma with or without MG.

**Methods:** Molecular profiles of thymoma with or without MG were downloaded from The Cancer Genome Atlas, and Pearson’s correlation analysis was performed to identify immune-related lncRNAs. T test was used to examine the differential expression and differential methylation between thymoma patients with or without MG. Gene Ontology and Kyoto Encyclopedia of Genes and Genomes analyses were performed to predict the function of target genes of immune-related lncRNAs.

**Results:** Analyses of the 87 thymoma samples with complete MG information revealed that 205 mRNAs and 56 lncRNAs showed up-regulated expression in thymoma with MG patients, while 458 mRNAs and 84 lncRNAs showed down-regulated expression. The methylation level of three immune-related lncRNAs (AP000787.1, AC004943.1, WT1-AS, FOXG1-AS1) was significantly decreased in thymoma tissues, and the methylation level of these immune-related lncRNAs (WT1-AS: Cor = 0.368, *p* < 0.001; FOXG1-AS1: Cor = 0.288, *p* < 0.01; AC004943.1: Cor = -0.236, *p* < 0.05) correlated with their expression. GO and KEGG pathway analysis revealed that targets of the immune-related lncRNA FOXG1-AS1 were enriched in small GTPase binding and herpes simplex virus 1 infection. Transcription coregulator activity and cell cycle were the most enriched pathways for targets of lncRNA AC004943.1. LncRNA WT1-AS targets were most enriched in actin binding and axon guidance.

**Conclusion:** Our results revealed the immune-related molecular profiling of thymoma with MG and without MG and identified key pathways involved in the underlying molecular mechanism of thymoma-related MG. These findings provide insights for further research of potential markers for thymoma-related MG.

## Introduction

Thymoma is the most common anterior mediastinal tumor in adults, and approximately 50% of thymoma patients show myasthenia gravis (MG) (Garibaldi et al., 2020). Thymectomy is effective for thymoma-associated MG, but the symptoms of some patients remain the same or become worse after surgical treatment (Kato et al., 2020). Although a link between thymoma and MG has long been established, the cause of thymoma-related MG remains elusive. For example, even among patients with similar age, sex, social-economic situation and histological types, some thymoma patients show MG while others do not show MG. Therefore, genetic factors may contribute to the development of MG-associated thymoma.

MG is an autoimmune disease associated with thymus abnormalities that are characterized by rapid skeletal muscle fatigue (Kumar, 2015). Thymoma-related MG is associated with abnormalities of the thymus, an important lymphoid organ that is closely related to immunity. Immune-related genes play an important function in the progression of tumors and these immune-related proteins remodel the tumor microenvironment through synergistic effects with other cytokines (Raica, Cimpean, & Ribatti, 2008; Chen, Qin, & Liu, 2020). Thymoma-related MG is usually more severe compared with generalized disease and is associated with bulbar and respiratory symptoms (Evoli et al., 2002). Previous studies showed that thymoma-related MG is likely mediated by dysfunctions in immunoregulation (Shelly, Agmon-Levin, Altman, & Shoenfeld, 2011; Bernard et al., 2016).

Long noncoding RNAs (lncRNAs) function in many biological processes, including transcriptional regulation and cell differentiation (Ponting, Oliver, & Reik, 2009), and play vital roles in cancer immunity (Yu, Wang, He, Xu, & Wang, 2018). Research has identified several lncRNAs implicated in MG (Barzago et al., 2016), and several aberrantly expressed lncRNAs were found in MG with thymoma (Luo et al., 2015; Niu, Jiang, Yin, & Hu, 2020). Hypomethylation and hypermethylation are common mechanisms underlying the aberrant expression of lncRNAs, which further impacts the regulation of their target genes. However, few studies have investigated the regulatory mechanisms of lncRNAs in thymoma-associated MG compared with thymoma without MG. Further understanding of the roles of immune-related lncRNAs in the molecular mechanism of thymoma patients with MG will be of great significance in the management of patients in the clinic.

Our current study aimed to investigate the potential underlying molecular mechanism of immune-related lncRNAs in the pathogenesis of thymoma-related MG using The Cancer Genome Atlas (TCGA) dataset. We investigated the differential expression and methylation profiles of thymoma patients with MG and without MG.

## Materials and Methods

### Data Collection

DNA methylation data of 94 thymoma cases (normal: n = 2; tumor: n = 92) and RNA-seq of 92 cases (normal: n = 2; tumor: n = 90) were downloaded from TCGA (https://portal.gdc.cancer.gov/). The clinical data of the patients were also downloaded. Human Genome Annotated Data (GRCh38) were used to annotate to gene ID (https://asia.ensembl.org/index.html). Immune-related gene sets IMMUNE_RESPONSE (Immune response M19817) and IMMUNE_SYSTEM_PROCESS (Immune system process M13664) were downloaded from the Gene Set Enrichment Analysis (GSEA) (http://www.gsea-msigdb.org/gsea/index.jsp).

### Identification of Immune-Related Genes and Immune-Related lncRNAs

Pearson’s correlation analysis was performed on immune-related genes and lncRNA expression levels in samples to identify immune-related lncRNAs according to the correlation coefficients and *p* values (|correlation coefficient|>0.6 and *p* < 0.001).

### Identification of Differential Expression and Differential Methylation

R package (edgeR) was used to identify the differential expression of lncRNAs (fold change (FC) > 2, adjusted *p* < 0.05) and differential methylation of lncRNAs (logFC filter>0.5, adjusted *p* < 0.05) between thymoma patients with MG and those without MG. Methylation sites of immune-related lncRNAs were identified according to GRCh38.

### Function Prediction of Immune-Related lncRNAs

The target genes of lncRNAs were identified from Pearson’s correlation analysis (|correlation coefficient|>0.4 and *p* < 0.001). Gene Ontology (GO) and Kyoto Encyclopedia of Genes and Genomes (KEGG) analyses were performed to predict the functions of the target genes of the immune-related lncRNAs (*p* < 0.01).

### Statistical Analysis

Chi-square test was used to analyze the differences in clinical information between thymoma patients with MG and those without MG. R packages (limma and edgeR) were used to identify differential expression and differential methylation. Bioconductor analyses (“org.Hs.eg.db,” “DOSE,” “pathview” and “clusterProfiler”) were performed to predict the function of immune-related lncRNAs. Data were analyzed using STATA/MP version 15.0 (StataCorp, College Station, TX, United States) and R software version 4.0.4 for Windows 10.

## Results

### Demographic Characteristics of the Patients

We obtained DNA methylation and RNA-seq data from thymoma cases from TCGA, and 90 thymoma tumor tissues had complete methylation and RNA-seq information. We excluded three samples that were missing information on MG history. A total of 87 patients were included in our study, including 29 patients with thymoma with MG and 58 patients with thymoma without MG. Comparison of patient characteristics between the two groups revealed no differences in age, gender, race, history of malignancy and Masaoka stage (Table 1). However, we found a significant difference in the distribution of WHO classification (*p* < 0.01).

**TABLE 1:** Patient characteristics of the two groups of thymoma patients.

Characteristics	Patient group, n (%)		*χ* ^*2*^	*P*
	Thymoma with MG (n = 29)	Thymoma without MG (n = 58)		
Age (n = 87)			1.472	0.225
≤60 years	17 (58.6)	26 (44.8)		
>60 years	12 (41.4)	32 (55.2)		
Gender (n = 87)			0.092	0.762
Male	14 (48.3)	30 (51.7)		
Female	15 (51.2)	28 (48.3)		
Race (n = 86)			2.573	0.276
White	24 (85.7)	45 (77.6)		
Asian	4 (14.3)	8 (13.8)		
Black	0 (0.0)	5 (8.6)		
History of malignancy (n = 87)			1.243	0.265
No	28 (96.5)	52 (89.7)		
Yes	1 (3.5)	6 (10.3)		
WHO classification (n = 87)			15.281	0.009
A	3 (10.3)	8 (13.8)		
AB	6 (20.7)	20 (34.5)		
B1	2 (6.9)	8 (13.8)		
B2	13 (44.8)	9 (15.5)		
B3	5 (17.2)	4 (6.9)		
C	0 (0.0)	9 (15.5)		
Masaoka stage (n = 87)			4.947	0.422
Ⅰ	8 (27.6)	19 (33.3)		
Ⅱa	12 (41.4)	23 (40.4)		
Ⅱb	5 (17.2)	5 (8.8)		
Ⅲ	3 (10.3)	7 (12.3)		
Ⅳa	1 (3.5)	0 (0.0)		
Ⅳb	0 (0.0)	3 (5.3)		

### Differential Expression of mRNAs and lncRNAs

**FIGURE 1:**
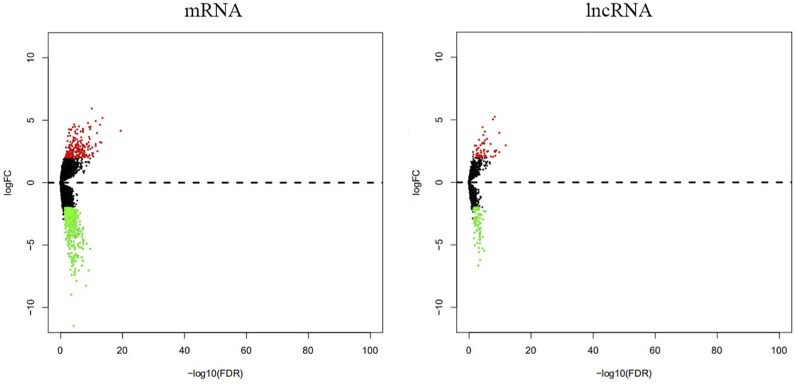
Differential expression of mRNAs and lncRNAs between thymoma with MG and thymoma without MG.

Our analyses identified 205 mRNAs and 56 lncRNAs that were up-regulated in thymoma with MG patients, while 458 mRNAs and 84 lncRNAs were down-regulated (Supplementary Table S1, Table2 and Figure 1). Among the differentially expressed genes, there were 10 immune-related genes (up-regulated expression: n = 2; down-regulated expression: n = 8) and 112 immune-related lncRNAs (up-regulated expression: n = 40; down-regulated expression: n = 72). There were 51 pairs of immune-related mRNA-lncRNA identified in the differential expression genes. The CCL20, CD24, DEFB4A, LTF, IL12A, CXCL13, and PYDC1 mRNAs and their co-expressed immune-related lncRNAs showed down-regulated expression in thymoma patients with MG compared with thymoma patients without MG, while IL31RA mRNA and its co-expression immune-related lncRNAs showed up-regulated expression (Table 2).

**TABLE 2:** Differentially expressed immune-related gene pairs.

mRNA			lncRNA			Correlation coefficient	*P*
name	logFC		name	logFC			
CCL20	−7.018592748	down	HOXC-AS1	−3.468290741	down	0.698	0.000
CCL20	−7.018592748	down	AL691482.3	−4.331371618	down	0.668	0.000
CCL20	−7.018592748	down	AC011700.1	−4.090216284	down	0.688	0.000
CCL20	−7.018592748	down	DLGAP1-AS5	−5.027264466	down	0.602	0.000
CCL20	−7.018592748	down	AC124248.1	−2.012883266	down	0.716	0.000
CCL20	−7.018592748	down	NAALADL2-AS2	−4.863497438	down	0.813	0.000
CCL20	−7.018592748	down	HOXC-AS2	−3.774436195	down	0.726	0.000
CCL20	−7.018592748	down	AC013640.1	−3.160517281	down	0.667	0.000
CD24	−3.828638114	down	CASC8	−2.116268906	down	0.909	0.000
CD24	−3.828638114	down	AC023886.1	−5.253853447	down	0.917	0.000
CD24	−3.828638114	down	AC078993.1	−2.874110729	down	0.924	0.000
CD24	−3.828638114	down	LL22NC03-63E9.3	−2.04428945	down	0.744	0.000
CD24	−3.828638114	down	AC012123.1	−2.072013437	down	0.625	0.000
CD24	−3.828638114	down	AL691482.3	−4.331371618	down	0.882	0.000
CD24	−3.828638114	down	AC025580.2	−2.620992792	down	0.948	0.000
CD24	−3.828638114	down	AP001150.1	−3.073247043	down	0.831	0.000
CD24	−3.828638114	down	AC025580.1	−5.291768385	down	0.932	0.000
DEFB4A	−6.61740337	down	AC104365.1	−3.042337304	down	0.987	0.000
DEFB4A	−6.61740337	down	TMEM132D-AS1	−6.646550893	down	0.993	0.000
DEFB4A	−6.61740337	down	LSAMP-AS1	−2.021421143	down	0.909	0.000
DEFB4A	−6.61740337	down	EGFLAM-AS4	−4.460946345	down	0.992	0.000
DEFB4A	−6.61740337	down	SLCO4A1-AS1	−3.962515293	down	0.941	0.000
LTF	−3.712976488	down	AC104365.1	−3.042337304	down	0.980	0.000
LTF	−3.712976488	down	TMEM132D-AS1	−6.646550893	down	0.984	0.000
LTF	−3.712976488	down	LSAMP-AS1	−2.021421143	down	0.935	0.000
LTF	−3.712976488	down	EGFLAM-AS4	−4.460946345	down	0.981	0.000
LTF	−3.712976488	down	SLCO4A1-AS1	−3.962515293	down	0.959	0.000
IL12A	−2.200880413	down	AC022762.1	−2.219385719	down	0.791	0.000
IL12A	−2.200880413	down	AL160191.1	−2.442168709	down	0.844	0.000
IL12A	−2.200880413	down	PCAT7	−2.96040145	down	0.798	0.000
IL12A	−2.200880413	down	AP000526.1	−2.008528258	down	0.786	0.000
IL12A	−2.200880413	down	AC100823.1	−2.789794422	down	0.771	0.000
IL12A	−2.200880413	down	LINC01587	−2.011010842	down	0.814	0.000
IL12A	−2.200880413	down	AL137804.1	−2.516876723	down	0.788	0.000
CXCL13	−2.267538905	down	AC104248.1	−2.176317368	down	0.647	0.000
CXCL13	−2.267538905	down	AC106882.1	−2.009902912	down	0.710	0.000
PYDC1	−2.267538905	down	FAM222A-AS1	−4.157505836	down	0.959	0.000
PYDC1	−2.267538905	down	CASC19	−2.953486218	down	0.826	0.000
PYDC1	−2.267538905	down	AC022509.1	−2.379085229	down	0.954	0.000
PYDC1	−2.267538905	down	AC013640.1	−3.160517281	down	0.655	0.000
IL31RA	2.700590226	up	AC005532.1	2.407791325	up	0.651	0.000
IL31RA	2.700590226	up	AC055874.1	3.475440443	up	0.620	0.000
IL31RA	2.700590226	up	AL392089.1	2.435856003	up	0.608	0.000
IL31RA	2.700590226	up	AC004160.2	2.165213075	up	0.603	0.000
IL31RA	2.700590226	up	AC079467.1	3.40186293	up	0.666	0.000
IL31RA	2.700590226	up	AL035425.1	2.26370864	up	0.610	0.000
IL31RA	2.700590226	up	AC010547.2	2.122136548	up	0.610	0.000
IL31RA	2.700590226	up	AF131216.3	2.344830265	up	0.653	0.000
IL31RA	2.700590226	up	LINC01341	2.442281038	up	0.704	0.000
IL31RA	2.700590226	up	AC025284.1	2.14237812	up	0.651	0.000
IL31RA	2.700590226	up	AL035425.3	2.554999873	up	0.650	0.000

### Differential DNA Methylation of Immune-Related lncRNAs

**FIGURE 2:**
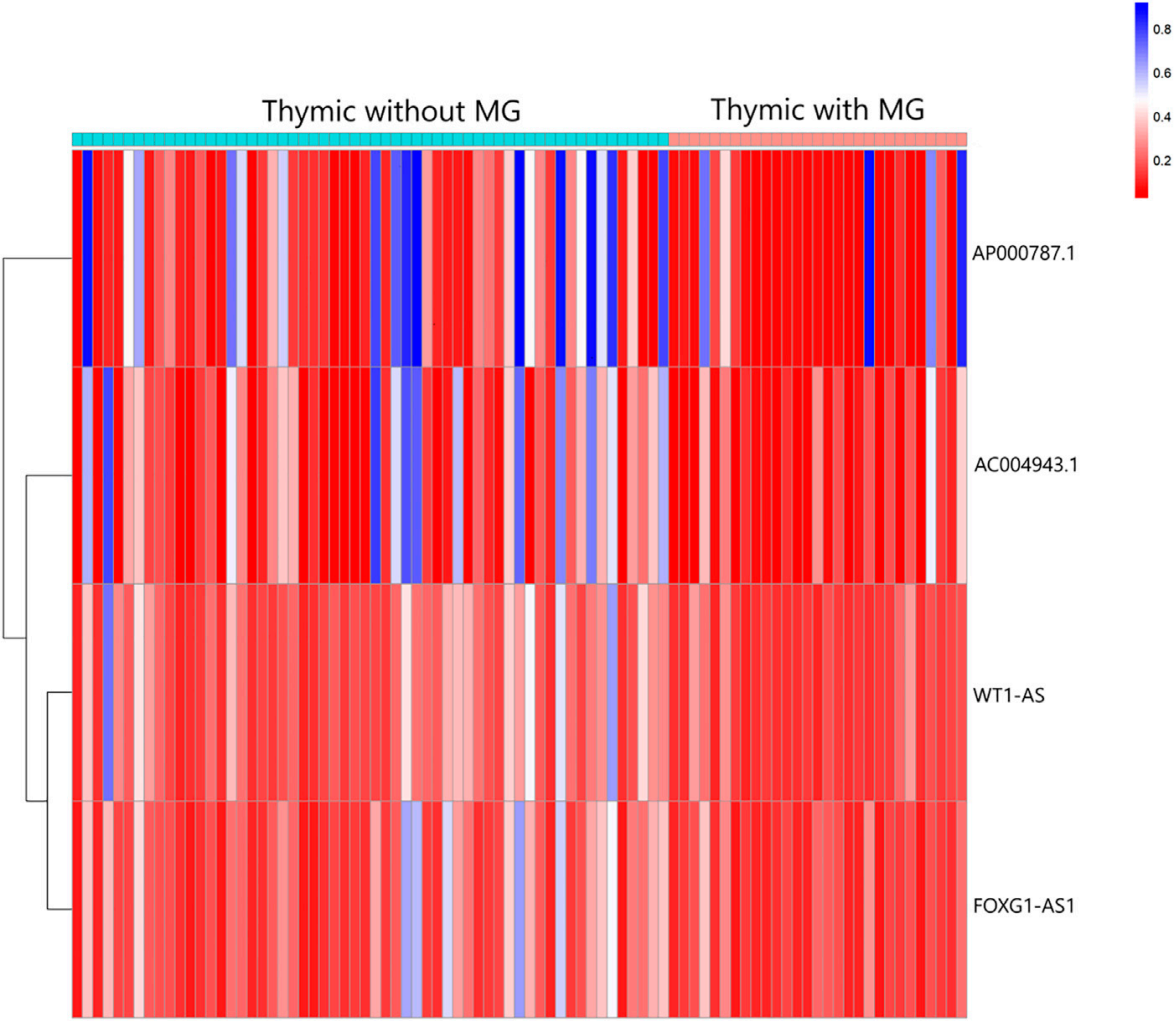
Heatmap analysis of DNA methylation in immune-related lncRNAs in patients with thymoma with MG and patients without MG.

**FIGURE 3:**
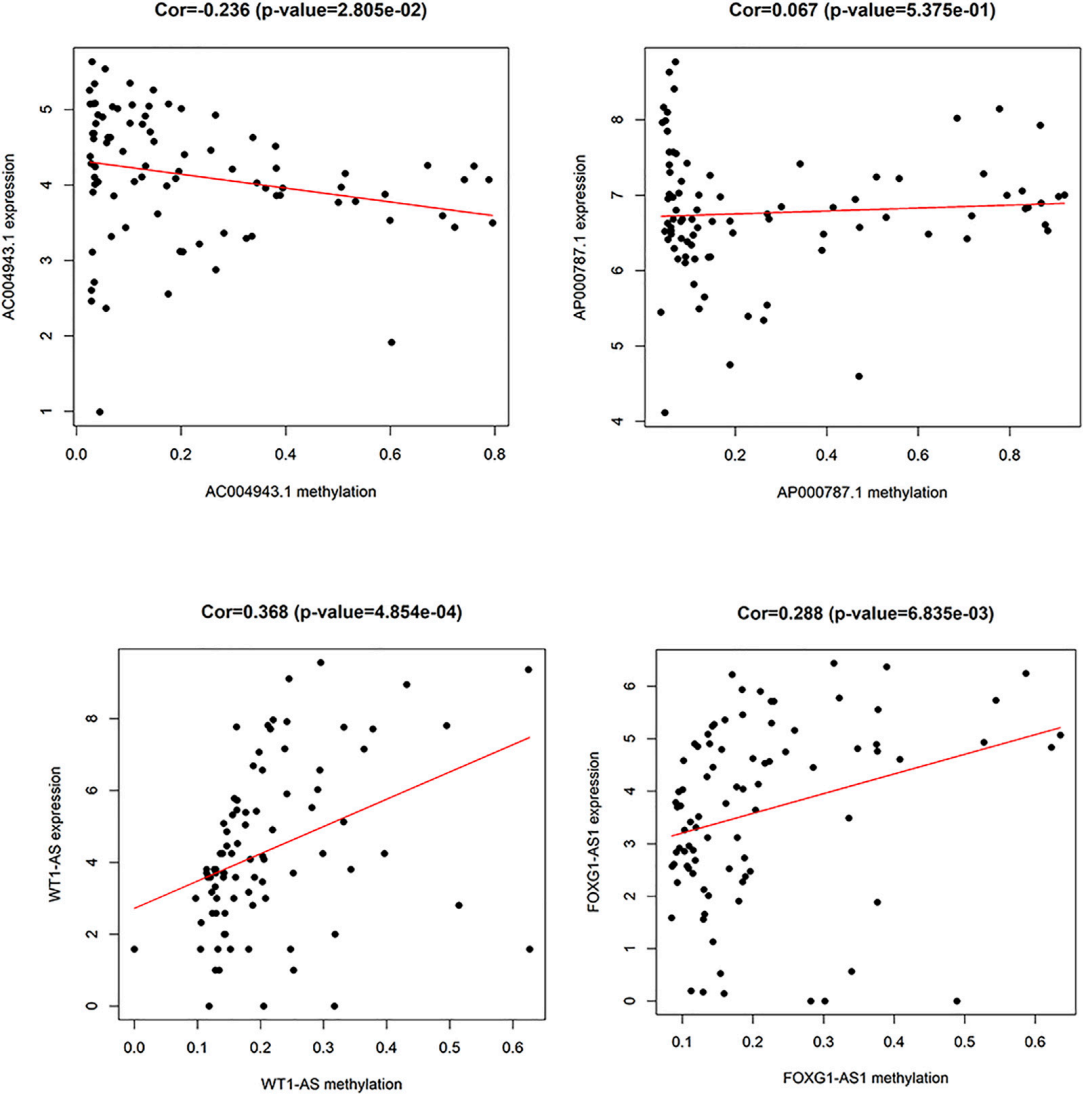
Relationship of the expression level and DNA methylation level of immune-related lncRNAs.

A total of 74 differential DNA methylation sites were found between the thymoma with MG and thymoma without MG patients, and four differential DNA methylation sites were found in immune-related lncRNAs (AC004943.1, FOXG1-AS1, WT1-AS, and AP000787.1) (Figure 2). The expression of lncRNAs WT1-AS (Cor = 0.368, *p* < 0.001) and FOXG1-AS1 (Cor = 0.288, *p* < 0.01) showed positive correlations with the methylation level, while higher expression of the lncRNA AC004943.1 was correlated with a lower DNA methylation level (Cor = -0.236, *p* < 0.05) (Figure 3).

### Function of the Immune-Related lncRNAs

**FIGURE 4:**
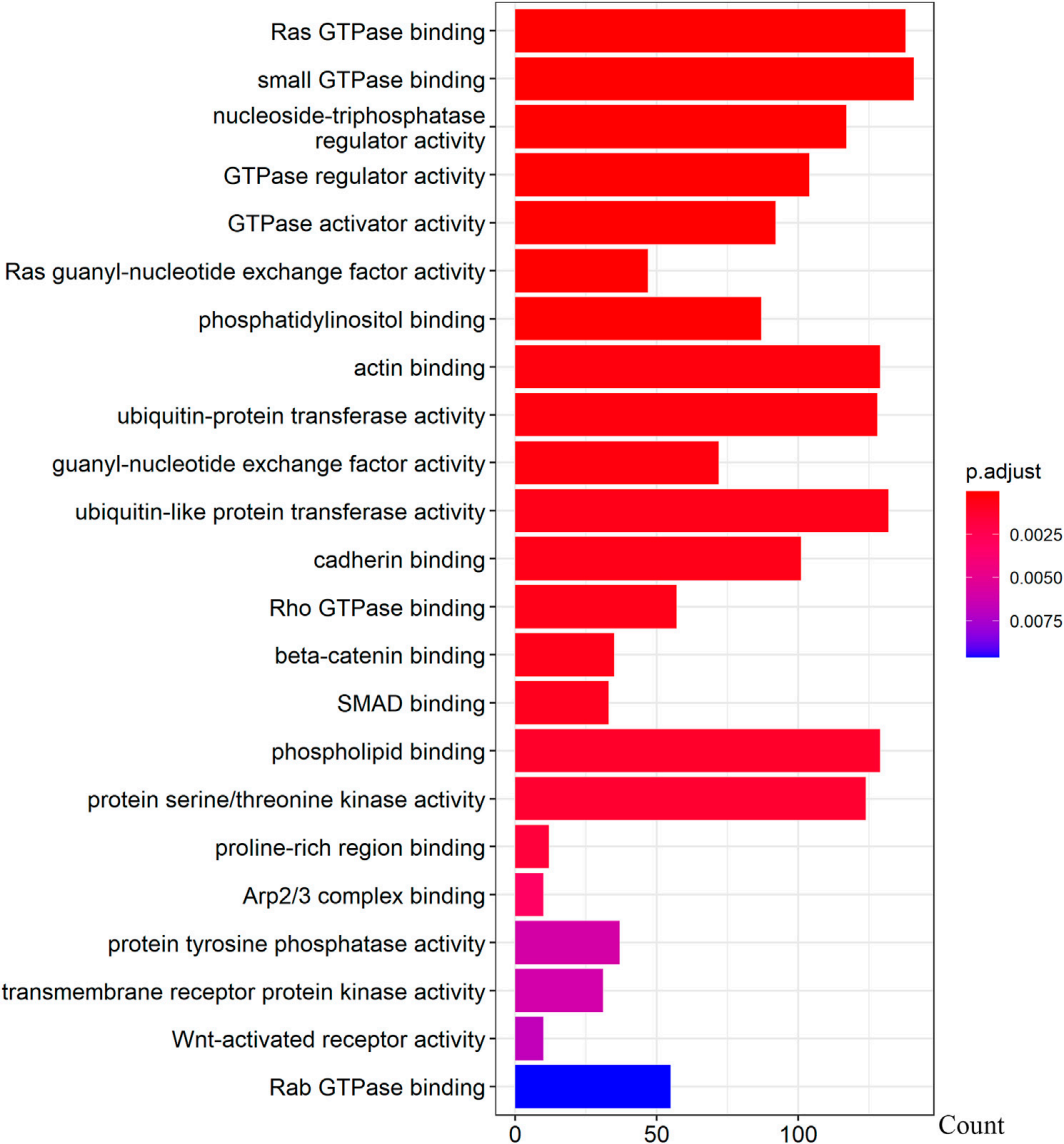
GO analysis of FOXG1-AS1 target genes. Note: The target genes of lncRNAs were identified from Pearson’s correlation analysis (|correlation coefficient|>0.4 and *p* < 0.001), GO analysis were performed in “clusterProfiler” and “org.Hs.eg.db” of R software to generated these functional pathways.

**FIGURE 5:**
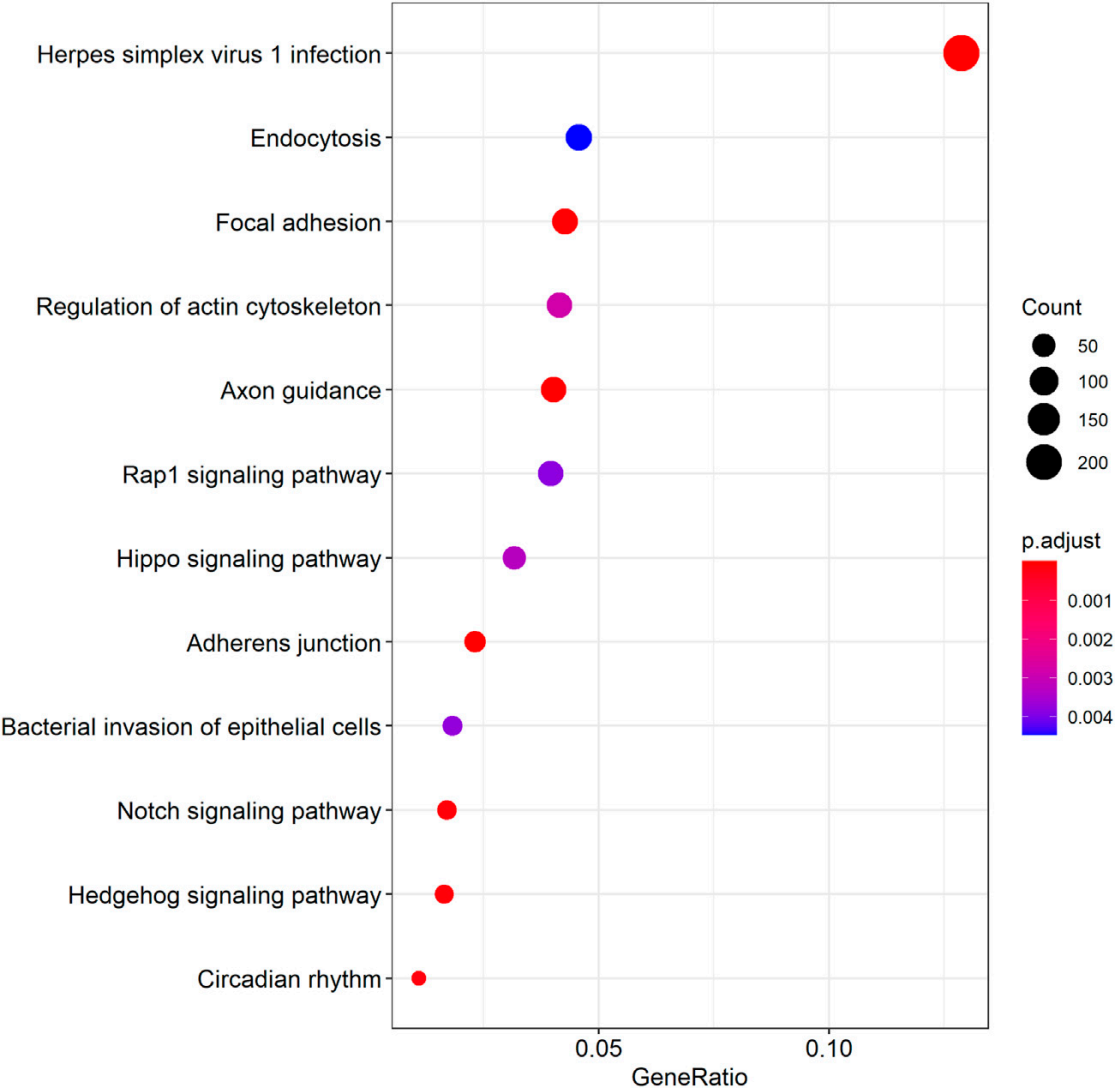
KEGG analysis of FOXG1-AS1 target genes. Note: The target genes of lncRNAs were identified from Pearson’s correlation analysis (|correlation coefficient|>0.4 and *p* < 0.001), KEGG analysis were performed in “clusterProfiler” and “DOSE” of R software to generated these functional pathways.

**FIGURE 6:**
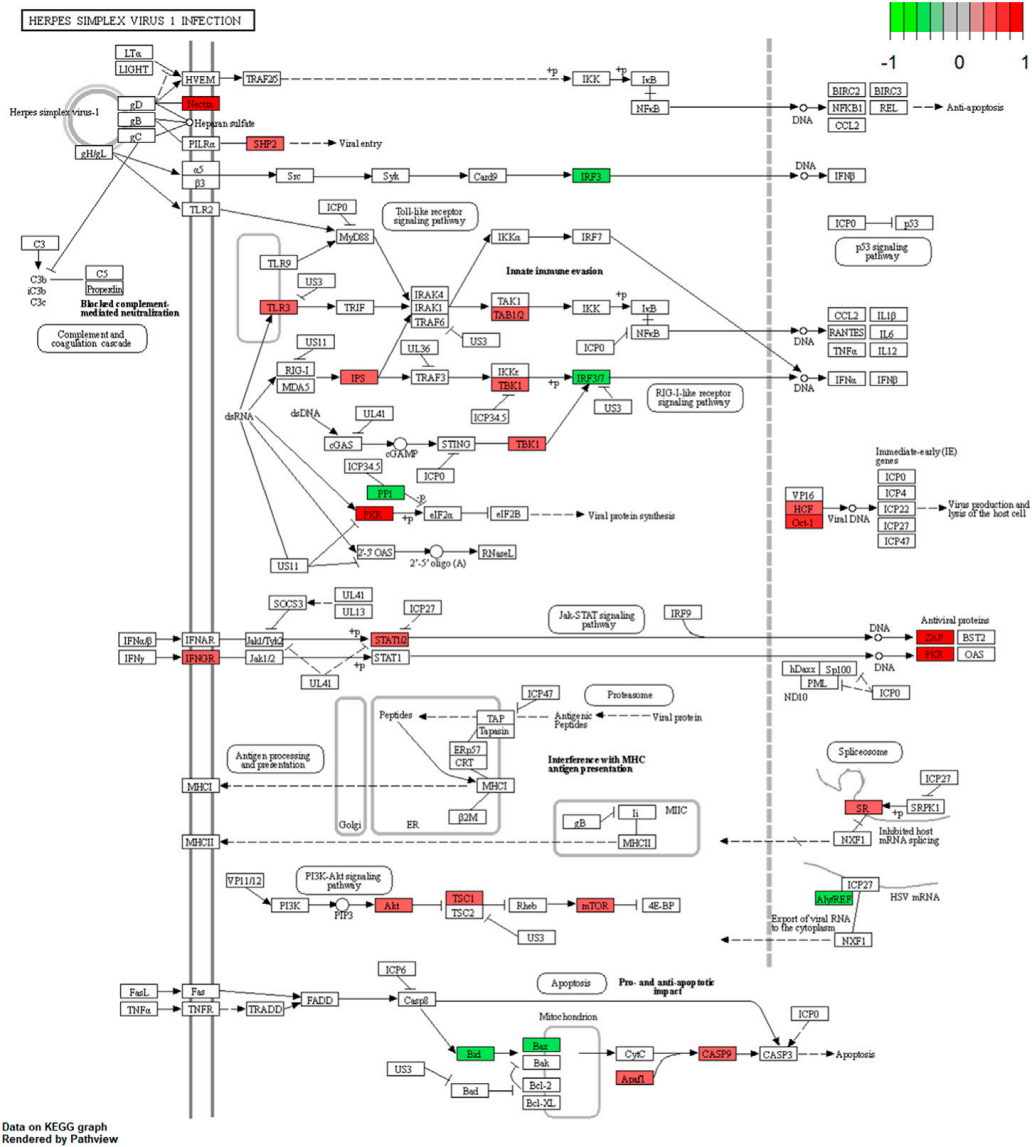
Pathway of herpes simplex virus 1 infection. Note: Visualization of a pathway significantly enriched by KEGG analysis and performed in “pathview” of R software.

**FIGURE 7:**
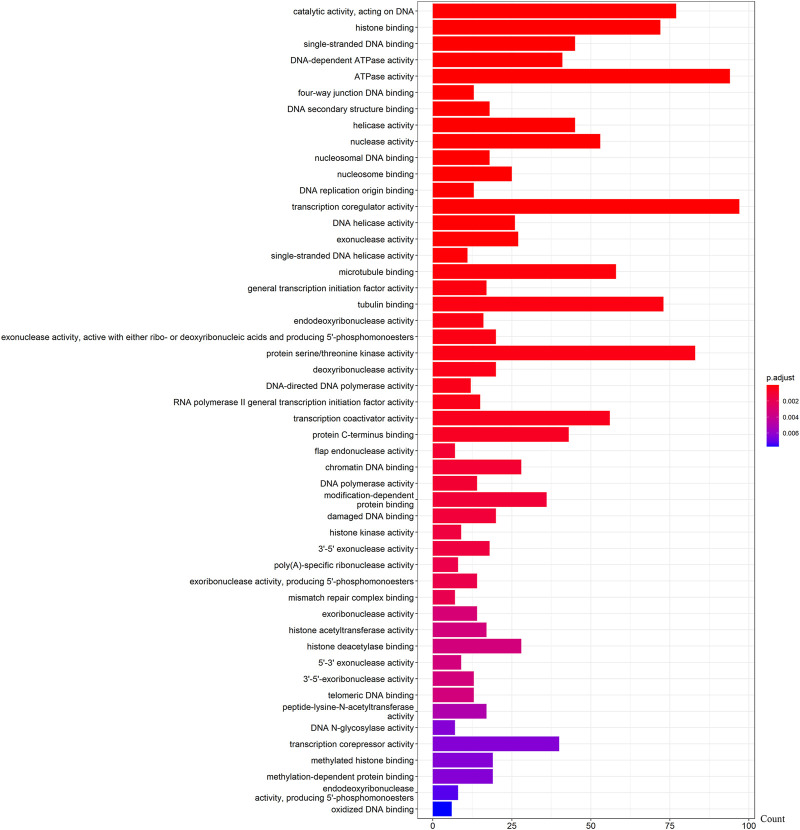
GO analysis of AC004943.1 target genes. Note: The target genes of lncRNAs were identified from Pearson’s correlation analysis (|correlation coefficient|>0.4 and *p* < 0.001), GO analysis were performed in “clusterProfiler” and “org.Hs.eg.db” of R software to generated these functional pathways.

**FIGURE 8:**
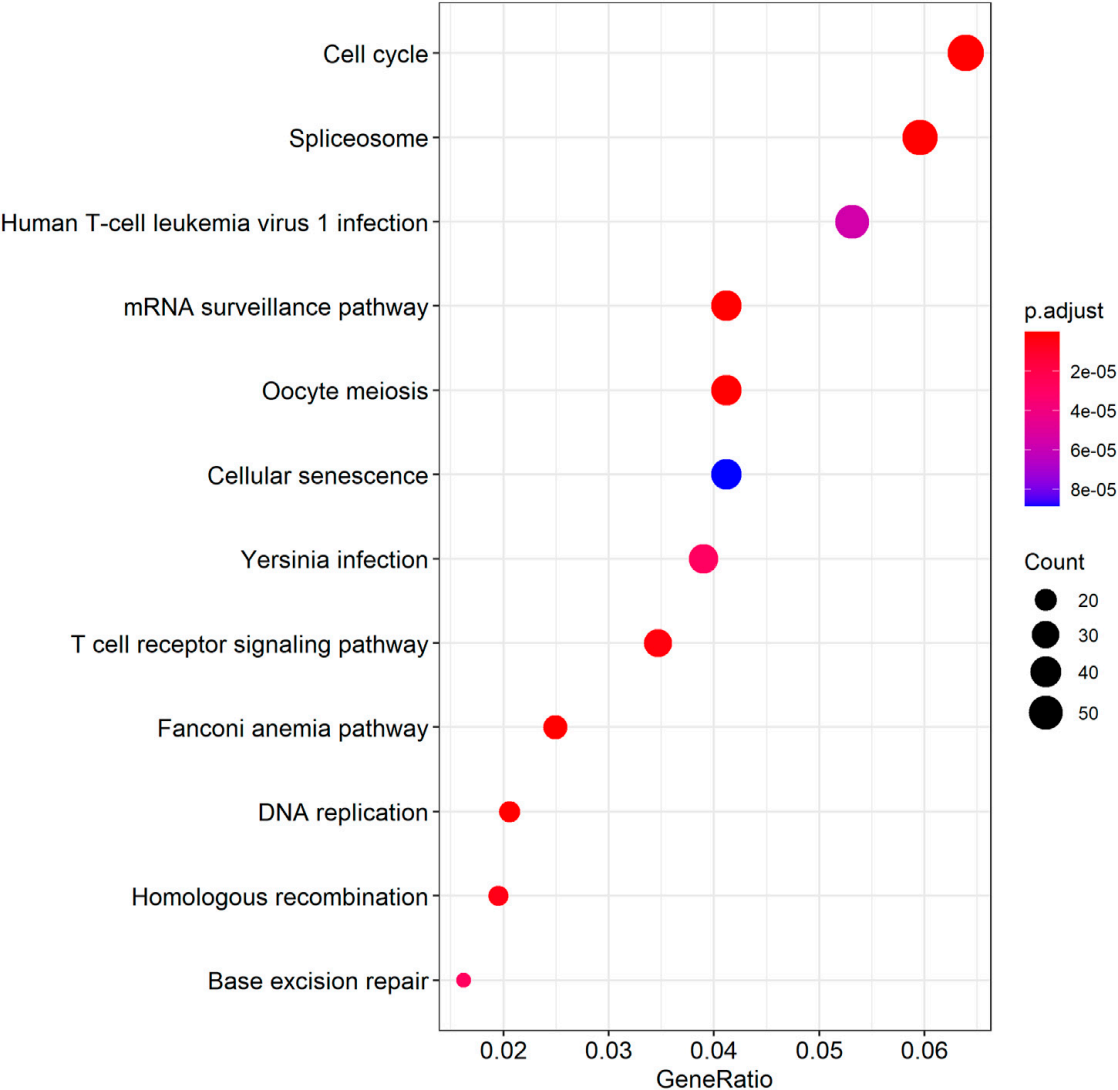
KEGG analysis of AC004943.1 target genes. Note: The target genes of lncRNAs were identified from Pearson’s correlation analysis (|correlation coefficient|>0.4 and *p* < 0.001), KEGG analysis were performed in “clusterProfiler” and “DOSE” of R software to generated these functional pathways.

**FIGURE 9:**
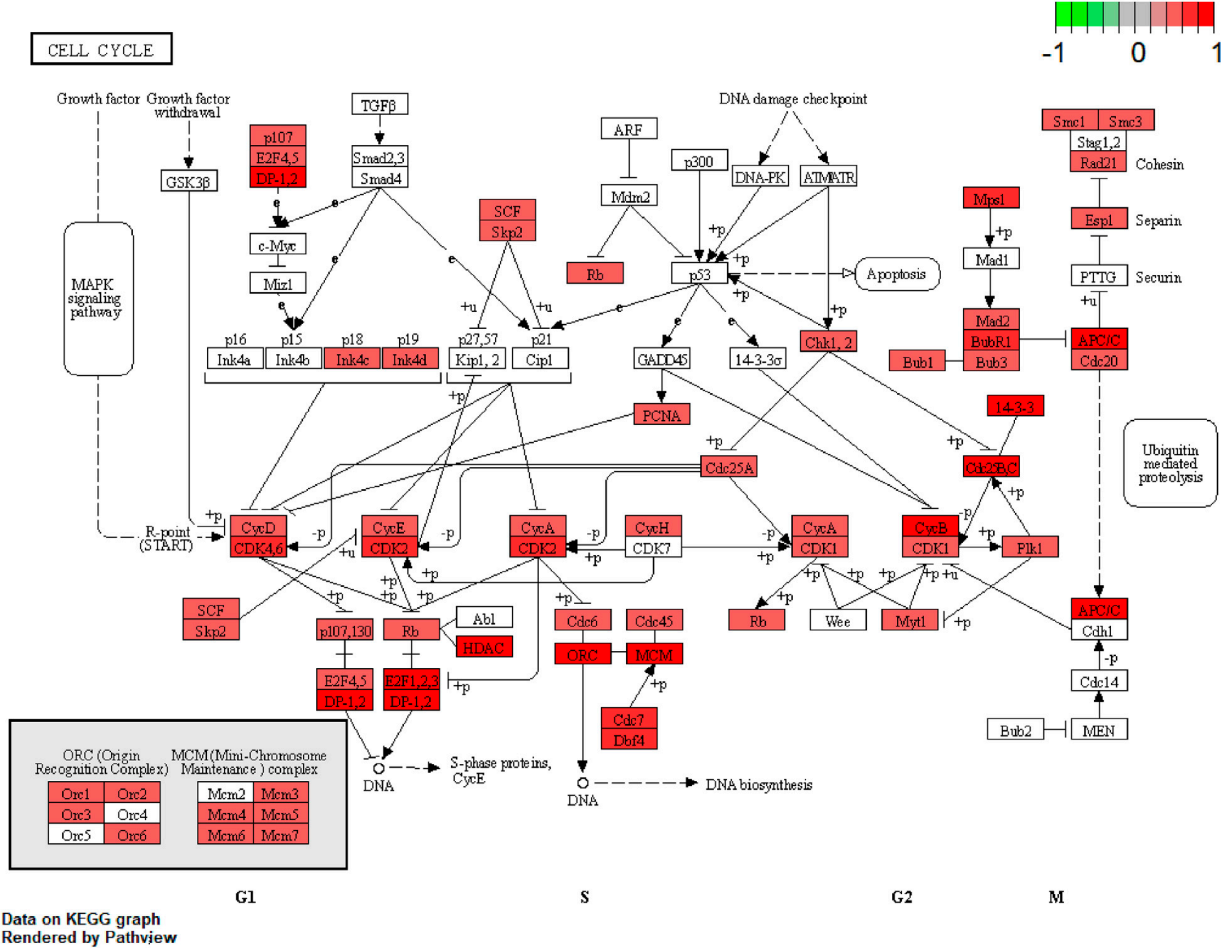
Pathway of cell cycle. Note: Visualization of a pathway significantly enriched by KEGG analysis and performed in “pathview” of R software.

**FIGURE 10:**
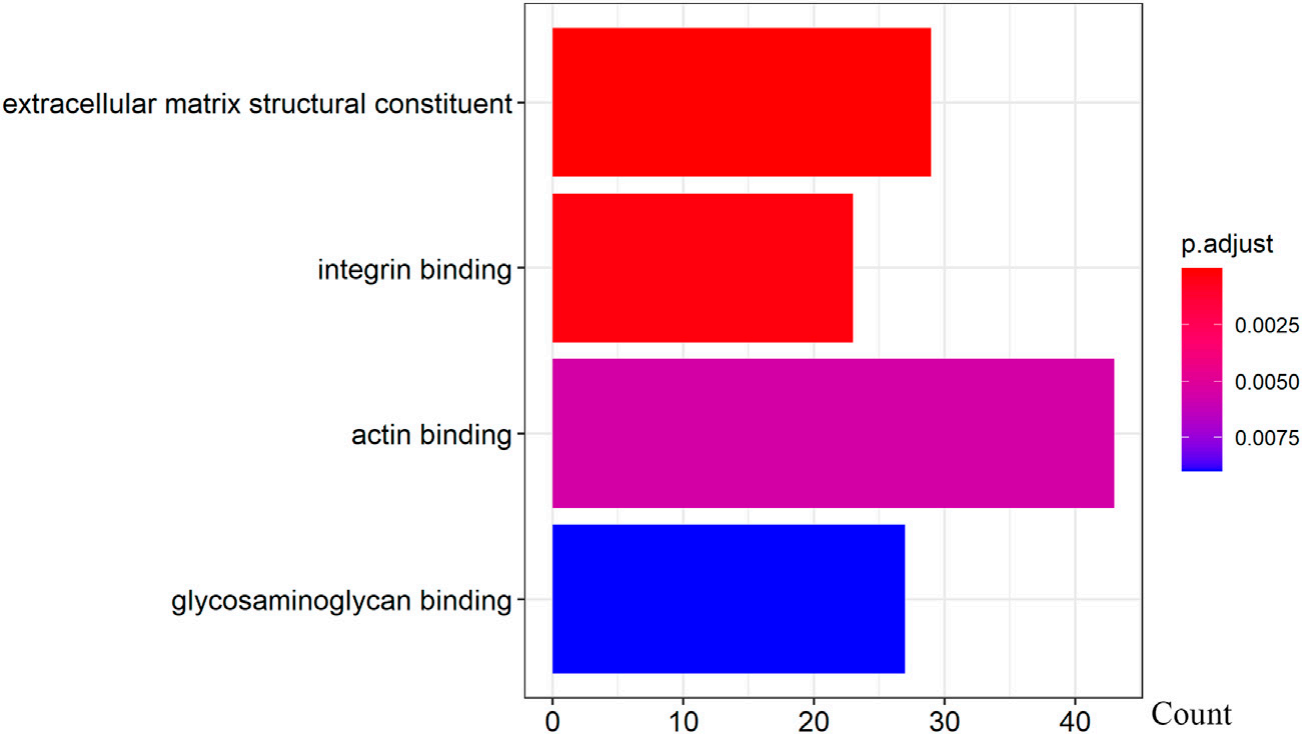
GO analysis of WT1-AS target genes. Note: The target genes of lncRNAs were identified from Pearson’s correlation analysis (|correlation coefficient|>0.4 and *p* < 0.001), GO analysis were performed in “clusterProfiler” and “org.Hs.eg.db” of R software to generated these functional pathways.

**FIGURE 11:**
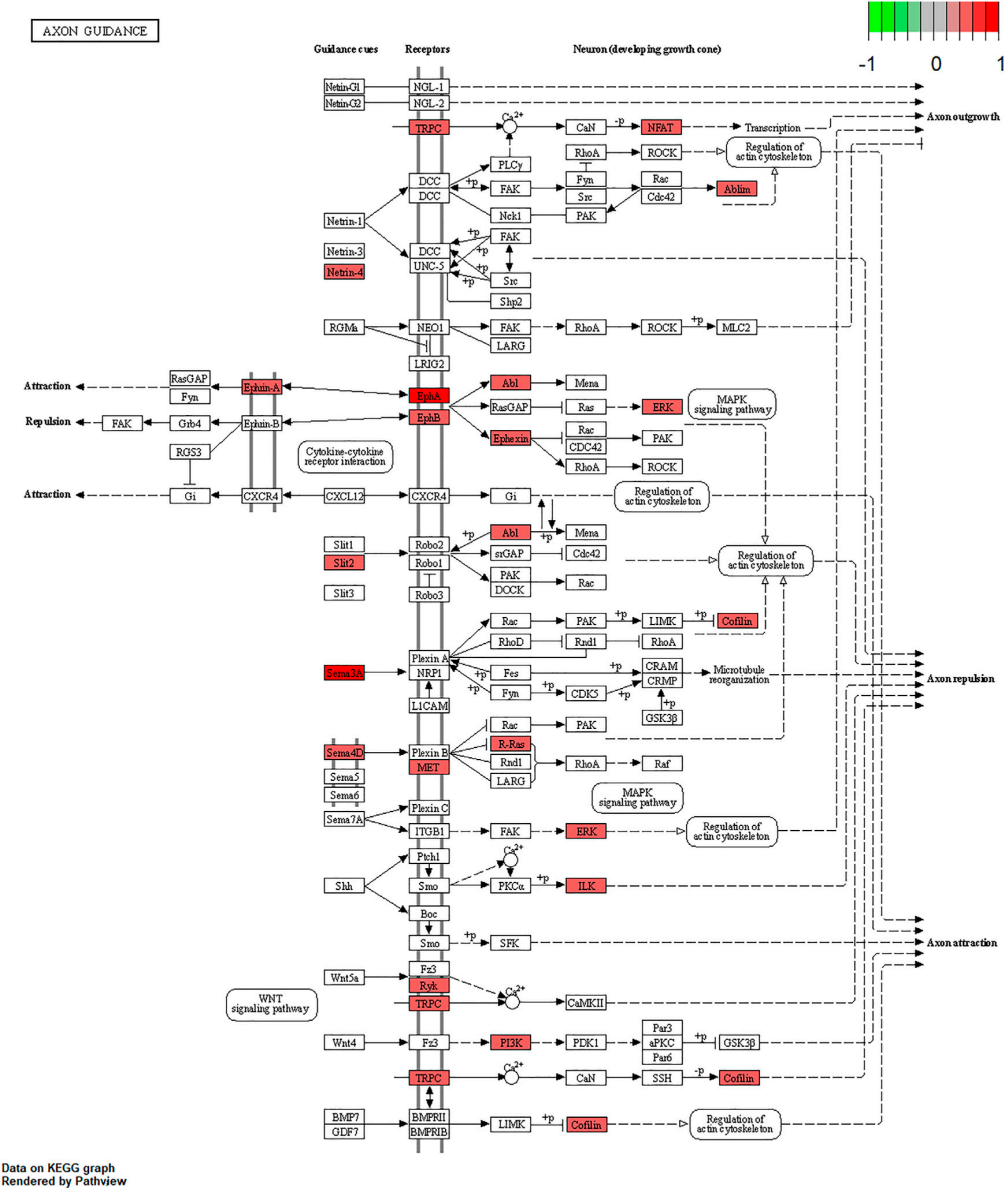
Pathway of axon guidance. Note: Visualization of a pathway significantly enriched by KEGG analysis and performed in “pathview” of R software.

We further examined the three immune-related lncRNAs (AC004943.1, FOXG1-AS1, WT1-AS) that showed a correlation with methylation level to predict the function of these immune-related lncRNAs that showed differential DNA methylation levels in thymoma with MG patients. GO analysis showed that the target genes of FOXG1-AS1 were most enriched in small GTPase binding, and KEGG analysis showed that herpes simplex virus 1 infection was the most enriched pathway (Figure 4, Figure 5, Figure 6). The target genes of lncRNA AC004943.1 were mostly enriched in transcription coregulator activity in GO analysis, and the most enriched pathway was cell cycle in KEGG analysis (Figure 7, Figure 8, Figure 9). Actin binding and axon guidance were the most enriched pathway of the lncRNA WT1-AS in GO analysis and KEGG analysis (Figure 10, Figure 11).

## Discussion

Previous studies have demonstrated an association between MG and thymoma; however, the pathogenesis of thymoma-related MG has remained elusive. In the present study, we explored the immune-related molecular profiling of thymoma with MG and without MG. We found that a series of immune-related genes and lncRNAs were differently expressed in thymoma with and without MG. Our study identified four differential DNA methylation sites in immune-related lncRNAs (AC004943.1, FOXG1-AS1, WT1-AS and AP000787.1). We further discovered that the methylation levels of immune-related lncRNAs AC004943.1, FOXG1-AS1 and WT1-AS regulated the expression of these lncRNAs, GO and KEGG analyses revealed the functional pathways of the target genes of these immune-related lncRNAs.

CCL20, a member of CC family and the alpha subfamily chemokines, was found down-expressed in thymoma associated MG in our exploration. CCL20 was important in autoimmune disease. According to previous study, an increase of T lymphocytes may have a link to thymoma-related MG (Mishra et al., 2021), while the capacity to activate T lymphocytes were different in dendritic cells that induced by different level of CCL20 (Thomachot et al., 2004). CD24 was recognized as a genetic risk factor of multiple autoimmune disease (Tan, Zhao, Xiang, Chang, & Lu, 2016). In addition, our results also identified that the expression of CD24 is related to MG. The findings about different expression of inflammatory gene such as IL12A and IL31RA in thymoma with or without MG further confirmed the evidence that the incidence of MG may associated to infection. As Milan Radovich’s study provided, genetic alterations were have a link to thymoma and MG (Radovich et al., 2018). Our finding further indicated that the expression of genes also alter in thymoma-related MG.

The Wilms’ tumor suppressor gene (WT1 gene) is located at 11p13, and WT1-AS is an antisense RNA that comprises 19–23 nucleotides and is complementary to WT1 (Xu, Zhang, & Zhang, 2018). A study identified an antisense regulatory region in intron 1 of the WT1 gene, located on chromosome 11p13, in Wilms’ tumors as a primary site for epigenetic deregulation (Malik et al., 2000). WT1-AS regulates its target, the WT1 gene, by binding to the TATA region of the WT1 gene promotor (Lv, Chen, Zhou, Li, & Gong, 2015). In this study, we found that DNA methylation in the promoter region of the lncRNA WT1-AS was significantly decreased in thymoma-related MG tissues compared with thymoma without MG and resulted in modulation of lncRNA expression levels, which may influence the expression of downstream genes that are related to the development of thymoma-related MG.

A clinical trial conducted in Japan reported that WT1-specific immune responses were observed in the majority of thymoma patients (Oji et al., 2018). However, the specific regulatory mechanism of WT1 in thymoma-related MG is not clear. Recent studies reported that WT1-AS is involved in the development of many diseases through its regulation of the expression of specific target genes (Le, Luo, Ouyang, & Zhong, 2020; Qiu et al., 2020; Wang, Ge, Zhang, & Chen, 2020; Wan et al., 2021). To explore the function of lncRNA WT1-AS, we performed GO and KEGG enrichment analysis. GO analysis showed that the target genes of WT1-AS were enriched in actin binding, indicating it regulates the gene expression of proteins that bind actin and thereby potentially modulates the properties and/or functions of the actin filament. Axon guidance, a significantly enriched pathway found by KEGG analysis, allows the formation of intricate neural circuits that favor axons to form synaptic connections (Russell & Bashaw, 2018; Kim & Kim, 2020). Studies have shown that disrupted axon guidance contributes to neurological disorders (Niftullayev & Lamarche-Vane, 2019). As MG is an acquired autoimmune disease of nervous system that dysfunction of neuromuscular junction transmission, this result indicates that WT1-AS may function in the transmission of neuromuscular junctions and that its aberrant expression may contribute to the pathogenesis of thymoma-related MG.

FOXG1-AS1 is antisense RNA of the FOXG1 gene that is located at 14q12. Some evidence has shown that FOXG1 plays a key role in mediating cancer cell metastasis and radiosensitivity in tumors (Zheng et al., 2019; Xiao et al., 2021), but the functions of the antisense RNA FOXG1-AS1 have not been reported. Our results showed that the methylation level of lncRNA FOXG1-AS1 was lower in tumors of thymoma patients with MG, and its expression was related to the methylation level. Ras GTPases interact selectively and non-covalently with members of the Ras superfamily of monomeric GTPases. Our results identified Ras GTPase binding as one of the gene enrichment pathways of FOXG1-AS1 target genes. The Ras GTPase is a driver of many cancers, and some small GTPase proteins may bind alternative effectors that harbor Ras binding domains (Singh & Smith, 2020). Pathway analysis showed significant enrichment in herpes simplex virus 1 infection. Previous studies showed that recovery from a viral infection is regulated by the immune system (Wu, Wu, Shen, Jiang, & Xu, 2018). Persistent viral infection, especially infection from herpes simplex virus 1, is a contributing factor to some neuropathic diseases (Mangold & Szpara, 2019). In an etiological study of thymoma-associated MG, researchers found that MG might develop after pathogen infection (Cufi et al., 2014). Our results further indicated that the pathogenesis of thymoma-related MG may be associated with infection.

The methylation level of lncRNA AC004943.1 is lower in tumor tissues of thymoma patients with MG than those without MG, and its expression is down-regulated by a higher methylation level. GO and KEGG analysis revealed that the target genes of the immune-related lncRNA AC004943.1 regulated transcription coregulator activity and cell cycle. Deregulation of the cell cycle pathway is an important hallmark of cancer. Some studies investigated the association between cell cycle proteins and thymoma and found that cell cycle protein expression regulates the pathogenesis and influences the prognosis of thymoma (Baldi et al., 2005; Mineo, Ambrogi, Mineo, & Baldi, 2005; Mineo, Ambrogi, Baldi, Pompeo, & Mineo, 2009). Transcription coregulator activity modulates the transcription of specific gene sets by binding to DNA-bound transcription factors, and thus AC004943.1 may play a role in the occurrence of thymoma with MG by influencing regulators of transcription factors.

We identified three immune-related lncRNAs (AC004943.1, WT1-AS and FOXG1-AS1) which were alter in methylation and expression, and found the target genes of these immun-related lncRNAs were enriched in “regulated transcription coregulator activity”, “cell cycle”, “actin binding”, “axon guidance”, “Ras GTPase binding” and “herpes simplex virus 1 infection”. Previous study explored the functional pathways of thymoma and thymoma-related MG have found that differential expression of functional pathways were associated to MG in different thymoma histotypes (Yamada et al., 2020). Another recent study focus on the genetic characterization of thymoma found some signal pathways that enriched by the upregulated genes in thymoma with MG may be responsible for MG in thymoma patients (Yu, Ke, Du, Yu, & Gao, 2019). In order to provide a immune-related molecular profile of thymoma-associated MG, this present study mainly explored the functional pathways of immune-related lncRNAs that their methylation and expression level were connected to the thymoma patients with MG.

The predictive value of immune-related biomarkers associated thymoma-related MG remains controversial among different reports in the literature. Our present study identified immune-related lncRNA alterations to further the molecular understanding of the occurrence and development of thymoma-related MG and provided a set of immune-related genes and lncRNAs that may aid in identifying the key biomarkers that are responsible for thymoma-related MG. These findings present insights for further research into the pathogenesis of immune-related diseases.

This study has several limitations. First, the Gene Expression Omnibus database (https://www.ncbi.nlm.nih.gov/geo/) lacks expression data of thymoma and the current study only used the dataset downloaded from TCGA. Therefore, we were unable to obtain confirmatory data. In addition, some samples were excluded because of incomplete clinicopathological features of the history of MG, so the sample size was limited. The precise molecular functions of these immune-related lncRNAs have not yet been validated, and future studies are required to support our findings. Furthermore, TCGA dateset lack the information of antiacetylcholine antibody which was found correlated to thymoma-related MG, it’s valuable to explore the relationship between antiacetylcholine antibody and these immune-related lncRNAs in further studies.

## Conclusion

Our bioinformatics analysis of thymoma patients from TCGA dataset revealed a list of immune-related genes and lncRNAs for thymoma with MG. We found that the expression of immune-related mRNA-lncRNA pairs and methylation level of immune-related lncRNAs regulated the pathogenesis of thymoma-related MG. The methylation level of several immune-related lncRNAs (AC004943.1, WT1-AS, and FOXG1-AS1) correlated with the expression levels of these lncRNAs. The target genes of the lncRNAs may regulate the occurrence of thymoma-related MG through functional pathways related to the immune system and nervous system.

## Data Availability

Publicly available datasets were analyzed in this study. This data can be found here: https://CancerGenome.nih.gov/.
