# Supplementary material for: Immune-Related Molecular Profiling of Thymoma With Myasthenia Gravis
Source: Front Genet. 2021 Oct 28;12:756493. doi: 10.3389/fgene.2021.756493 (PMC8580862; doi:10.3389/fgene.2021.756493)
Supplement: Supplementary file 1 [file Table1.DOC]

| Supplementary Table 1 Differentially expressed mRNAs in thymoma with and without MG | | | | | |
| --- | --- | --- | --- | --- | --- |
| mRNA Name | logFC | logCPM | PValue | FDR | Expression in thymoma with MG |
|
| GLDC | 4.146203671 | 1.953152397 | 2.32E-24 | 4.14E-20 | up-regulated |
| NGB | 5.177780068 | 3.064828954 | 3.55E-18 | 3.18E-14 | up-regulated |
| BBOX1 | 3.203654354 | 2.713932757 | 1.19E-17 | 7.09E-14 | up-regulated |
| CRB1 | 3.244317366 | 0.912656986 | 4.78E-17 | 1.85E-13 | up-regulated |
| ONECUT3 | 4.631286106 | -1.680955918 | 5.16E-17 | 1.85E-13 | up-regulated |
| ENOX1 | 2.517417554 | 3.366346951 | 4.18E-16 | 1.25E-12 | up-regulated |
| VWC2 | 3.961013952 | 2.904717111 | 6.74E-16 | 1.72E-12 | up-regulated |
| EPHA5 | 4.927323989 | 1.377851861 | 2.03E-15 | 4.55E-12 | up-regulated |
| PTN | 3.085522462 | 6.390380736 | 2.63E-15 | 4.72E-12 | up-regulated |
| SGIP1 | 2.934658661 | 2.628011985 | 2.64E-15 | 4.72E-12 | up-regulated |
| LIPJ | 2.07682369 | -1.060632419 | 1.72E-14 | 2.79E-11 | up-regulated |
| TRIM7 | 2.345647999 | 3.557800813 | 2.33E-14 | 3.47E-11 | up-regulated |
| HR | 2.352646929 | 5.166781378 | 3.14E-14 | 4.33E-11 | up-regulated |
| TMEM163 | 2.815989075 | 5.199339459 | 4.37E-14 | 5.59E-11 | up-regulated |
| ALDH4A1 | 2.202199607 | 6.667031154 | 5.02E-14 | 5.99E-11 | up-regulated |
| MAP2 | 2.376837146 | 5.152129778 | 5.60E-14 | 6.26E-11 | up-regulated |
| GPR50 | 5.93454868 | -1.51019958 | 7.65E-14 | 8.05E-11 | up-regulated |
| KISS1 | 4.474910517 | 2.990580273 | 1.98E-13 | 1.97E-10 | up-regulated |
| RYR3 | 2.703223312 | 4.206004463 | 2.57E-13 | 2.42E-10 | up-regulated |
| FIBCD1 | 4.280805065 | 0.897178277 | 3.51E-13 | 2.86E-10 | up-regulated |
| CEND1 | 3.49136807 | 1.261373797 | 5.25E-13 | 4.09E-10 | up-regulated |
| CNKSR2 | 2.201542435 | 2.602387196 | 7.45E-13 | 5.12E-10 | up-regulated |
| FAM166A | 2.167664569 | -0.974487244 | 7.55E-13 | 5.12E-10 | up-regulated |
| THRSP | 4.446438706 | 0.447244233 | 7.72E-13 | 5.12E-10 | up-regulated |
| ACOT6 | 2.942688712 | -3.622481004 | 9.06E-13 | 5.79E-10 | up-regulated |
| SMIM32 | 3.653369517 | -0.404848423 | 9.76E-13 | 6.02E-10 | up-regulated |
| HGD | 3.227028681 | 1.86036205 | 1.41E-12 | 8.39E-10 | up-regulated |
| LRRC66 | 2.037382821 | -0.101435115 | 2.48E-12 | 1.34E-09 | up-regulated |
| IL13RA2 | 2.843193961 | 3.01225077 | 2.56E-12 | 1.34E-09 | up-regulated |
| PLXNB3 | 2.840322886 | 2.617048913 | 2.62E-12 | 1.34E-09 | up-regulated |
| SLITRK1 | 4.773784309 | 1.313336372 | 2.83E-12 | 1.41E-09 | up-regulated |
| TF | 2.631193386 | 2.449921674 | 2.91E-12 | 1.41E-09 | up-regulated |
| KLHDC8A | 3.038886391 | 1.901121025 | 6.49E-12 | 3.06E-09 | up-regulated |
| TRIM67 | 3.114166599 | 1.090585157 | 7.19E-12 | 3.30E-09 | up-regulated |
| VSIG1 | 2.124260784 | 1.315419682 | 8.56E-12 | 3.74E-09 | up-regulated |
| SLC22A4 | 2.179386296 | 2.016185581 | 1.03E-11 | 4.27E-09 | up-regulated |
| KBTBD12 | 2.230507915 | -1.46231655 | 1.18E-11 | 4.69E-09 | up-regulated |
| NPC1L1 | 2.210482811 | -2.376165285 | 3.11E-11 | 1.16E-08 | up-regulated |
| PDE1A | 2.846593528 | 4.254418491 | 3.33E-11 | 1.22E-08 | up-regulated |
| ASS1 | 2.034181674 | 6.890562426 | 4.64E-11 | 1.66E-08 | up-regulated |
| DNAH9 | 2.421122257 | 0.284242325 | 5.08E-11 | 1.78E-08 | up-regulated |
| EGF | 2.933104012 | -1.003258671 | 5.80E-11 | 1.97E-08 | up-regulated |
| LRRN3 | 2.471398701 | 3.728466583 | 7.16E-11 | 2.37E-08 | up-regulated |
| PPARGC1A | 2.147309852 | 4.284224481 | 8.01E-11 | 2.60E-08 | up-regulated |
| TCTEX1D1 | 2.555297149 | 0.602388501 | 8.14E-11 | 2.60E-08 | up-regulated |
| CAMK2B | 2.896383808 | 5.53304103 | 9.01E-11 | 2.83E-08 | up-regulated |
| TMEM63C | 2.06542838 | 0.756487691 | 1.26E-10 | 3.74E-08 | up-regulated |
| ANGPT4 | 2.294638991 | -0.662176186 | 1.36E-10 | 4.00E-08 | up-regulated |
| FYB2 | 3.008165621 | 2.097903877 | 1.68E-10 | 4.70E-08 | up-regulated |
| GABRE | 2.566375631 | 4.009342197 | 1.83E-10 | 5.05E-08 | up-regulated |
| MMP10 | 4.182223793 | 2.578498982 | 2.25E-10 | 5.93E-08 | up-regulated |
| CSMD3 | 4.063220929 | 0.190083953 | 2.53E-10 | 6.30E-08 | up-regulated |
| LIPF | 4.313889494 | -1.07423964 | 2.53E-10 | 6.30E-08 | up-regulated |
| SMPX | 2.971292295 | -2.088004559 | 2.87E-10 | 7.05E-08 | up-regulated |
| ADAMTS18 | 2.351710227 | 3.759947445 | 3.43E-10 | 7.97E-08 | up-regulated |
| RBP5 | 2.236705366 | 3.375756396 | 3.54E-10 | 8.11E-08 | up-regulated |
| ANKRD55 | 2.12231328 | 1.213853054 | 3.68E-10 | 8.35E-08 | up-regulated |
| VEGFD | 2.767330143 | 1.187912907 | 3.89E-10 | 8.61E-08 | up-regulated |
| SHISA6 | 2.714875303 | -1.526842242 | 3.89E-10 | 8.61E-08 | up-regulated |
| VSTM2L | 2.222963443 | 3.101036565 | 4.01E-10 | 8.75E-08 | up-regulated |
| FAM135B | 2.933233665 | -0.35731905 | 4.06E-10 | 8.75E-08 | up-regulated |
| MEGF11 | 3.054174867 | 4.099121972 | 4.38E-10 | 9.23E-08 | up-regulated |
| TRPC5OS | 2.716984578 | -2.258048669 | 7.15E-10 | 1.41E-07 | up-regulated |
| PHF24 | 2.53610968 | 1.783140385 | 7.92E-10 | 1.52E-07 | up-regulated |
| RGS4 | 2.612122284 | 2.184731943 | 8.35E-10 | 1.57E-07 | up-regulated |
| GLRA4 | 3.384881234 | 0.546984604 | 8.52E-10 | 1.59E-07 | up-regulated |
| SYNDIG1L | 2.430752336 | -0.366801668 | 1.09E-09 | 1.93E-07 | up-regulated |
| ASB4 | 2.013084266 | 0.353012582 | 1.26E-09 | 2.17E-07 | up-regulated |
| GUCY2F | 3.619749296 | 0.871491942 | 1.30E-09 | 2.22E-07 | up-regulated |
| ATP2B3 | 3.192484161 | 0.146535987 | 1.54E-09 | 2.61E-07 | up-regulated |
| ITLN1 | 4.094263154 | 0.605152927 | 2.04E-09 | 3.31E-07 | up-regulated |
| PCP4L1 | 2.899681522 | 3.575356428 | 2.92E-09 | 4.54E-07 | up-regulated |
| OSBPL6 | 2.018444742 | 2.378448623 | 3.38E-09 | 5.13E-07 | up-regulated |
| FAM189A1 | 3.352218284 | 1.015696597 | 3.38E-09 | 5.13E-07 | up-regulated |
| SYT7 | 2.316467733 | 2.202789089 | 3.42E-09 | 5.14E-07 | up-regulated |
| CTAGE9 | 2.604845007 | -2.637411883 | 4.49E-09 | 6.43E-07 | up-regulated |
| FAM205C | 2.635443951 | -1.947900777 | 4.73E-09 | 6.71E-07 | up-regulated |
| KRT32 | 2.308704294 | 2.909635176 | 4.76E-09 | 6.71E-07 | up-regulated |
| RBFOX3 | 2.451403307 | 0.752657538 | 4.90E-09 | 6.84E-07 | up-regulated |
| CYP21A2 | 2.00814444 | 2.661653518 | 5.16E-09 | 7.10E-07 | up-regulated |
| FAM71F1 | 2.699460255 | -2.578129055 | 5.94E-09 | 8.12E-07 | up-regulated |
| SLC35G3 | 2.056854232 | -2.833404963 | 8.12E-09 | 1.08E-06 | up-regulated |
| S100A7 | 4.509977855 | -0.286492714 | 8.32E-09 | 1.09E-06 | up-regulated |
| JPH3 | 2.18639948 | -1.287588116 | 9.09E-09 | 1.19E-06 | up-regulated |
| GRM3 | 2.242090547 | -1.356175867 | 1.02E-08 | 1.32E-06 | up-regulated |
| CA1 | 2.940225211 | -2.914211207 | 1.49E-08 | 1.82E-06 | up-regulated |
| ALDH1A1 | 2.138156737 | 5.007429377 | 1.53E-08 | 1.85E-06 | up-regulated |
| SEC14L6 | 2.785404738 | -0.059033073 | 1.71E-08 | 2.04E-06 | up-regulated |
| PCSK1 | 2.522161017 | 1.035307685 | 1.80E-08 | 2.13E-06 | up-regulated |
| DPYS | 3.035475256 | 4.069061923 | 1.84E-08 | 2.15E-06 | up-regulated |
| FBXW12 | 2.108654579 | -1.631899588 | 1.88E-08 | 2.19E-06 | up-regulated |
| SCRG1 | 3.264873063 | 0.825905131 | 1.90E-08 | 2.19E-06 | up-regulated |
| POU3F1 | 2.134427482 | 1.984970237 | 2.07E-08 | 2.33E-06 | up-regulated |
| RNF128 | 2.473892387 | 3.678572072 | 2.41E-08 | 2.63E-06 | up-regulated |
| OR13J1 | 2.649735108 | -2.672997067 | 2.43E-08 | 2.63E-06 | up-regulated |
| ABCC12 | 3.038602201 | -3.032439902 | 2.48E-08 | 2.67E-06 | up-regulated |
| C2orf66 | 2.151898314 | -0.593529316 | 3.39E-08 | 3.49E-06 | up-regulated |
| LRTM2 | 3.901483213 | 4.545032187 | 3.81E-08 | 3.81E-06 | up-regulated |
| OR2A4 | 3.132728999 | -3.07406339 | 3.82E-08 | 3.81E-06 | up-regulated |
| CLDN19 | 2.759783797 | 1.655201648 | 3.84E-08 | 3.81E-06 | up-regulated |
| NEFM | 3.453112874 | 5.435211401 | 4.43E-08 | 4.20E-06 | up-regulated |
| ISX | 4.251556152 | -2.334730216 | 4.44E-08 | 4.20E-06 | up-regulated |
| IGFBP1 | 2.538213604 | -0.725506152 | 5.21E-08 | 4.83E-06 | up-regulated |
| C9orf92 | 2.2088778 | -3.031253239 | 5.80E-08 | 5.27E-06 | up-regulated |
| ENPP3 | 2.447625564 | 5.383502231 | 6.39E-08 | 5.69E-06 | up-regulated |
| VAT1L | 2.797154285 | 0.382216817 | 6.54E-08 | 5.76E-06 | up-regulated |
| GRIA3 | 2.758108567 | -1.232480605 | 7.63E-08 | 6.54E-06 | up-regulated |
| TCERG1L | 2.1258692 | 3.807120695 | 8.33E-08 | 6.93E-06 | up-regulated |
| PTCHD1 | 3.017704771 | 1.510541555 | 8.78E-08 | 7.24E-06 | up-regulated |
| CYP4F3 | 3.036701831 | 2.392297829 | 1.34E-07 | 1.04E-05 | up-regulated |
| USP26 | 2.415419622 | -2.273116276 | 1.42E-07 | 1.08E-05 | up-regulated |
| NPBWR1 | 2.890473169 | -3.22245063 | 1.58E-07 | 1.19E-05 | up-regulated |
| GBP6 | 3.005631127 | 1.555582303 | 1.92E-07 | 1.42E-05 | up-regulated |
| HOXA13 | 3.964783504 | -1.05212857 | 2.19E-07 | 1.57E-05 | up-regulated |
| EVX1 | 3.559341789 | -2.515164891 | 2.47E-07 | 1.75E-05 | up-regulated |
| FGA | 4.109190916 | -3.429596845 | 2.58E-07 | 1.80E-05 | up-regulated |
| KRT15 | 2.4655656 | 8.739855492 | 2.60E-07 | 1.80E-05 | up-regulated |
| HSPA2 | 2.065148546 | 6.343778554 | 3.11E-07 | 2.12E-05 | up-regulated |
| MC3R | 4.476888866 | -3.413085543 | 3.34E-07 | 2.23E-05 | up-regulated |
| LHFPL4 | 3.395607862 | 0.091172712 | 3.93E-07 | 2.52E-05 | up-regulated |
| SPOCK1 | 2.800544964 | 6.185066137 | 5.46E-07 | 3.29E-05 | up-regulated |
| PDYN | 4.66370773 | 0.870720097 | 7.38E-07 | 4.28E-05 | up-regulated |
| KLHL1 | 3.571789218 | -0.582721712 | 7.45E-07 | 4.29E-05 | up-regulated |
| IL31RA | 2.700590226 | 2.207778906 | 1.09E-06 | 5.87E-05 | up-regulated |
| CCL19 | 2.126395447 | 8.118330986 | 1.15E-06 | 6.08E-05 | up-regulated |
| ZDHHC22 | 3.329762427 | -2.440016644 | 1.16E-06 | 6.09E-05 | up-regulated |
| SLC28A1 | 2.067481303 | -1.458048119 | 1.49E-06 | 7.42E-05 | up-regulated |
| SFTPC | 3.630133064 | 4.90368692 | 1.52E-06 | 7.48E-05 | up-regulated |
| RBFOX1 | 2.575280174 | -1.735461798 | 1.55E-06 | 7.61E-05 | up-regulated |
| GABRA5 | 2.64946119 | 4.429419413 | 1.72E-06 | 8.26E-05 | up-regulated |
| IRS4 | 3.420836506 | 3.578936293 | 1.77E-06 | 8.42E-05 | up-regulated |
| PLEKHD1 | 2.443539906 | 3.130289434 | 1.95E-06 | 9.05E-05 | up-regulated |
| PRSS38 | 2.287930768 | -3.729686903 | 2.13E-06 | 9.59E-05 | up-regulated |
| MGAT4C | 2.473135035 | 0.52081914 | 2.32E-06 | 0.000102729 | up-regulated |
| LCE3E | 4.440290713 | -3.612828267 | 2.50E-06 | 0.00010892 | up-regulated |
| RBMXL3 | 3.257489096 | -3.751735016 | 2.73E-06 | 0.000116435 | up-regulated |
| MROH7-TTC4 | 2.165212592 | -2.16474339 | 2.86E-06 | 0.000120078 | up-regulated |
| HSD3B2 | 2.395870749 | -1.485881348 | 4.26E-06 | 0.00016615 | up-regulated |
| KRT6C | 2.91393104 | 2.711011511 | 4.51E-06 | 0.000172245 | up-regulated |
| INSYN2B | 2.497812292 | 0.107480451 | 5.78E-06 | 0.000208488 | up-regulated |
| SORCS3 | 2.248113423 | -3.869190356 | 5.99E-06 | 0.000213078 | up-regulated |
| TCEAL6 | 2.052557335 | -3.684593179 | 6.14E-06 | 0.000217262 | up-regulated |
| RAX2 | 2.610435895 | -1.872301356 | 6.75E-06 | 0.000234601 | up-regulated |
| FGG | 3.106242777 | -3.03662368 | 7.62E-06 | 0.000256404 | up-regulated |
| CXorf49B | 2.547789277 | -3.851174305 | 8.77E-06 | 0.000286053 | up-regulated |
| CLCA2 | 2.24981831 | 3.41642957 | 8.82E-06 | 0.000287143 | up-regulated |
| SPRR2G | 4.264122894 | -2.04084513 | 1.12E-05 | 0.000351825 | up-regulated |
| FGF10 | 2.343597923 | 2.041932848 | 1.13E-05 | 0.000354098 | up-regulated |
| NOS1 | 2.346753617 | -0.126032363 | 1.34E-05 | 0.000402554 | up-regulated |
| PGLYRP2 | 2.050539226 | -0.233301889 | 1.56E-05 | 0.000454428 | up-regulated |
| RTL4 | 2.674171629 | -3.879040095 | 1.71E-05 | 0.00048408 | up-regulated |
| AL035425.2 | 2.997744207 | -0.779113713 | 1.78E-05 | 0.000497515 | up-regulated |
| CRCT1 | 4.212857771 | -3.605107799 | 2.01E-05 | 0.000545992 | up-regulated |
| BCHE | 2.274732463 | 2.020211403 | 2.01E-05 | 0.000545992 | up-regulated |
| MYO18B | 2.320311564 | 2.996690114 | 2.15E-05 | 0.000572095 | up-regulated |
| PNPLA5 | 2.064599447 | -4.020158849 | 2.40E-05 | 0.000618227 | up-regulated |
| SFTPA2 | 3.261944014 | 3.753120013 | 2.65E-05 | 0.00066916 | up-regulated |
| CD300LD | 2.008093658 | -3.327910318 | 2.91E-05 | 0.000718023 | up-regulated |
| PCK1 | 2.629983675 | -0.464563121 | 2.99E-05 | 0.000730778 | up-regulated |
| EPHA8 | 2.34144899 | -3.133649743 | 3.15E-05 | 0.00076161 | up-regulated |
| SFTPA1 | 3.754587942 | 3.550014571 | 3.32E-05 | 0.000789765 | up-regulated |
| NEFL | 2.580797359 | 7.057646219 | 3.89E-05 | 0.000890298 | up-regulated |
| TRPC5 | 2.028116294 | -3.835454459 | 4.35E-05 | 0.000979684 | up-regulated |
| ITLN2 | 2.822291651 | 1.260016722 | 5.17E-05 | 0.001119465 | up-regulated |
| CYP11A1 | 2.07482289 | 1.349016026 | 5.52E-05 | 0.001179125 | up-regulated |
| DSG4 | 2.166188956 | -3.638972806 | 5.69E-05 | 0.001206143 | up-regulated |
| KRTDAP | 2.75962692 | -0.376636718 | 6.23E-05 | 0.001293534 | up-regulated |
| TMEFF2 | 2.104516815 | 2.55840692 | 6.23E-05 | 0.001293534 | up-regulated |
| SPHKAP | 2.457216053 | 2.570364827 | 6.35E-05 | 0.001311096 | up-regulated |
| SLITRK4 | 2.030323652 | 2.3379997 | 7.53E-05 | 0.001504199 | up-regulated |
| FSHB | 3.017157556 | -2.407067684 | 9.01E-05 | 0.001725421 | up-regulated |
| LCE3D | 3.994184676 | -2.451094981 | 9.63E-05 | 0.001814505 | up-regulated |
| RXFP2 | 4.278399317 | -2.382904463 | 0.000102655 | 0.001910115 | up-regulated |
| ATP10B | 2.251324856 | 5.275302021 | 0.000104087 | 0.001932738 | up-regulated |
| SBSN | 2.847366592 | 0.189128823 | 0.000117312 | 0.002116825 | up-regulated |
| PVALB | 2.01685685 | -1.515134168 | 0.000141478 | 0.002463486 | up-regulated |
| SOD3 | 2.117130673 | 6.101302443 | 0.000145983 | 0.002522298 | up-regulated |
| C14orf180 | 2.803714852 | 0.53027597 | 0.000152939 | 0.002624752 | up-regulated |
| MYOC | 2.455462798 | -1.900861454 | 0.000172262 | 0.002889874 | up-regulated |
| TMEM179 | 2.263164147 | -0.533357357 | 0.000208659 | 0.003316403 | up-regulated |
| HAO2 | 2.02482053 | 1.1489232 | 0.00028752 | 0.004230481 | up-regulated |
| CHST8 | 2.387519015 | 0.424815892 | 0.000311968 | 0.00447325 | up-regulated |
| SPRR2E | 3.733371213 | -2.780935826 | 0.000317969 | 0.004531567 | up-regulated |
| CLDN18 | 2.170209749 | -0.762031107 | 0.000326797 | 0.004638912 | up-regulated |
| ADGRD2 | 2.234962107 | 1.70477129 | 0.000401119 | 0.005406654 | up-regulated |
| DSG1 | 2.242564757 | 3.43731749 | 0.000409082 | 0.005489179 | up-regulated |
| OLIG2 | 3.326422889 | 0.435245144 | 0.000430971 | 0.005705909 | up-regulated |
| C4orf50 | 2.189375298 | 3.354412292 | 0.000449218 | 0.005899492 | up-regulated |
| NKX1-2 | 2.240900466 | -3.402559465 | 0.000462024 | 0.006023469 | up-regulated |
| PSORS1C2 | 2.05131181 | -1.962215814 | 0.00048988 | 0.006299458 | up-regulated |
| SYT4 | 2.467851209 | -2.311559073 | 0.000547567 | 0.006883039 | up-regulated |
| FGB | 2.416174577 | -3.389542372 | 0.000556527 | 0.006971186 | up-regulated |
| LRRC31 | 2.187077458 | -2.59340094 | 0.000636339 | 0.007688894 | up-regulated |
| CAPN6 | 2.511687155 | 2.322424807 | 0.000842666 | 0.009409683 | up-regulated |
| CHGB | 2.049721951 | 7.068727533 | 0.000865617 | 0.009535103 | up-regulated |
| SLITRK6 | 2.039328395 | 2.856096516 | 0.000916936 | 0.009911268 | up-regulated |
| MS4A12 | 2.118026459 | -3.304276986 | 0.001096364 | 0.011389967 | up-regulated |
| ADCY8 | 2.071167474 | -2.542432856 | 0.001318538 | 0.01301093 | up-regulated |
| PGC | 2.043805348 | -1.665237171 | 0.001389592 | 0.01352566 | up-regulated |
| KRT9 | 2.081451833 | 2.107300031 | 0.001547173 | 0.014653116 | up-regulated |
| OLIG1 | 2.27330412 | 2.833591656 | 0.001563242 | 0.014750675 | up-regulated |
| SCGB3A2 | 2.269994642 | -0.829441149 | 0.00191793 | 0.016970311 | up-regulated |
| SPRR2D | 2.817301417 | -1.81971116 | 0.002006854 | 0.017523743 | up-regulated |
| SFTA2 | 2.098332404 | -1.986089474 | 0.002022455 | 0.017642274 | up-regulated |
| LRRTM3 | 2.056786321 | -3.88004065 | 0.004199251 | 0.030431817 | up-regulated |
| GP2 | -11.46373663 | 5.226016162 | 1.06E-06 | 5.75E-05 | down-regulated |
| PRAC1 | -8.952034393 | -0.288527551 | 1.06E-05 | 0.000337228 | down-regulated |
| MYBPC1 | -8.263053294 | 6.367226309 | 1.69E-11 | 6.56E-09 | down-regulated |
| HOXB13 | -7.870563693 | 1.580264024 | 9.86E-08 | 7.99E-06 | down-regulated |
| LBX1 | -7.388595304 | 0.087546675 | 7.97E-06 | 0.00026565 | down-regulated |
| SP9 | -7.385560909 | -0.763596093 | 5.03E-07 | 3.07E-05 | down-regulated |
| NEUROG2 | -7.194700047 | -1.026451349 | 4.09E-07 | 2.58E-05 | down-regulated |
| TLX3 | -7.084066932 | -1.497070209 | 3.67E-07 | 2.39E-05 | down-regulated |
| C6orf222 | -7.030016634 | 1.303847419 | 1.08E-08 | 1.38E-06 | down-regulated |
| CCL20 | -7.018592748 | 4.298165775 | 1.54E-12 | 8.88E-10 | down-regulated |
| UMOD | -6.972446645 | -0.516628382 | 2.89E-05 | 0.000716188 | down-regulated |
| SLC18A3 | -6.891011276 | -1.552565024 | 2.66E-07 | 1.84E-05 | down-regulated |
| NKX2-3 | -6.692659122 | -0.567660675 | 5.98E-06 | 0.000213075 | down-regulated |
| MSLN | -6.633771524 | 6.395469199 | 6.84E-10 | 1.38E-07 | down-regulated |
| DEFB4A | -6.61740337 | 0.02058445 | 3.00E-06 | 0.000123688 | down-regulated |
| PRAC2 | -6.529969081 | -1.867007799 | 1.46E-06 | 7.30E-05 | down-regulated |
| UGT2A1 | -6.397800771 | -0.757430303 | 6.85E-06 | 0.00023684 | down-regulated |
| HTR3C | -6.303761622 | -1.564521301 | 1.86E-07 | 1.39E-05 | down-regulated |
| TMPRSS11E | -6.273054805 | 0.215377911 | 4.70E-07 | 2.91E-05 | down-regulated |
| UGT2B28 | -6.270721386 | -2.009200614 | 4.09E-06 | 0.000161455 | down-regulated |
| LCN1 | -6.239451265 | 0.865356038 | 1.97E-06 | 9.12E-05 | down-regulated |
| IGF2BP3 | -6.237563151 | 1.888663595 | 2.51E-10 | 6.30E-08 | down-regulated |
| NKX2-2 | -6.231181528 | 0.197880646 | 3.98E-07 | 2.52E-05 | down-regulated |
| CTAG2 | -6.123170685 | -2.529295393 | 8.29E-06 | 0.000274735 | down-regulated |
| C10orf99 | -6.055782723 | -0.238214484 | 1.26E-06 | 6.49E-05 | down-regulated |
| PLA2G3 | -6.018423015 | 0.442204456 | 3.11E-08 | 3.26E-06 | down-regulated |
| GRIA4 | -5.981536528 | 1.785843905 | 7.79E-10 | 1.52E-07 | down-regulated |
| STRA8 | -5.972735494 | 0.522556751 | 3.95E-07 | 2.52E-05 | down-regulated |
| MAGEA4 | -5.938552121 | 0.833244753 | 1.27E-05 | 0.000385518 | down-regulated |
| ZIC5 | -5.925099616 | -1.189951706 | 2.09E-06 | 9.50E-05 | down-regulated |
| HOXD11 | -5.909907642 | -1.717257079 | 3.52E-06 | 0.000141797 | down-regulated |
| FOXL2NB | -5.909534275 | -0.668687613 | 9.47E-05 | 0.001790597 | down-regulated |
| CDX2 | -5.892186535 | -1.248130356 | 7.83E-07 | 4.46E-05 | down-regulated |
| ZIC2 | -5.868765057 | -0.175368498 | 1.95E-08 | 2.24E-06 | down-regulated |
| KCNJ18 | -5.804939518 | -1.463190971 | 1.10E-07 | 8.71E-06 | down-regulated |
| MAGEA10 | -5.739357653 | -0.83003157 | 8.83E-05 | 0.001696853 | down-regulated |
| PLEKHG7 | -5.722669637 | 1.012995185 | 2.15E-07 | 1.54E-05 | down-regulated |
| BARX1 | -5.712774983 | 0.84240276 | 1.67E-07 | 1.26E-05 | down-regulated |
| DEFB4B | -5.670139111 | -2.724476615 | 0.000244933 | 0.00377306 | down-regulated |
| CCK | -5.638173774 | -1.254442905 | 9.73E-06 | 0.000312153 | down-regulated |
| MAGEB10 | -5.617282838 | -2.50292716 | 1.09E-05 | 0.000342672 | down-regulated |
| TRIM51GP | -5.325311369 | -1.768738566 | 4.56E-07 | 2.85E-05 | down-regulated |
| GFI1B | -5.297661401 | 2.607001443 | 1.92E-10 | 5.21E-08 | down-regulated |
| SOX2 | -5.291910809 | 2.902213306 | 3.28E-13 | 2.86E-10 | down-regulated |
| KLK3 | -5.277909452 | -2.02882023 | 3.96E-05 | 0.000901213 | down-regulated |
| TFAP2B | -5.271707745 | -0.451289141 | 3.00E-07 | 2.05E-05 | down-regulated |
| MMP3 | -5.2024843 | 3.012379842 | 2.52E-09 | 4.03E-07 | down-regulated |
| FUT6 | -5.190474519 | 0.404914357 | 1.06E-09 | 1.89E-07 | down-regulated |
| MAGEA9 | -5.184362037 | -3.340910971 | 8.66E-05 | 0.001670077 | down-regulated |
| TNR | -5.168507371 | 1.599393074 | 4.81E-07 | 2.97E-05 | down-regulated |
| ASCL3 | -5.168215327 | 0.366425648 | 6.24E-09 | 8.40E-07 | down-regulated |
| DHRS9 | -5.167056581 | 4.124648391 | 9.58E-10 | 1.75E-07 | down-regulated |
| SP8 | -5.112617773 | 0.616503163 | 1.52E-06 | 7.48E-05 | down-regulated |
| MYOG | -5.06766767 | -0.936702388 | 7.00E-06 | 0.000240605 | down-regulated |
| HTR3E | -5.05817691 | -1.131329754 | 1.42E-08 | 1.75E-06 | down-regulated |
| APOBEC1 | -5.009841523 | -3.01205431 | 1.24E-06 | 6.43E-05 | down-regulated |
| SOST | -5.004327383 | -0.032473678 | 1.63E-06 | 7.94E-05 | down-regulated |
| HOXD13 | -4.999566892 | -2.290002626 | 0.000259371 | 0.003917924 | down-regulated |
| TFF2 | -4.992206508 | -2.705957737 | 0.000134786 | 0.002378563 | down-regulated |
| MYL1 | -4.951799985 | -3.515510104 | 1.51E-06 | 7.48E-05 | down-regulated |
| OTX2 | -4.933600644 | -1.422273614 | 6.26E-05 | 0.001297735 | down-regulated |
| PROX1 | -4.915310219 | 2.281439832 | 1.42E-10 | 4.09E-08 | down-regulated |
| FOXI1 | -4.891640609 | 2.63734237 | 2.13E-08 | 2.36E-06 | down-regulated |
| KIT | -4.879189888 | 6.240080755 | 9.21E-12 | 3.93E-09 | down-regulated |
| NDST4 | -4.812739761 | -2.441962181 | 0.0002053 | 0.003281139 | down-regulated |
| HEPACAM2 | -4.808590722 | 3.031424905 | 3.68E-09 | 5.39E-07 | down-regulated |
| ARL14 | -4.772077546 | -0.761613275 | 1.46E-05 | 0.000429568 | down-regulated |
| HMX3 | -4.754152125 | -2.225917373 | 0.000141788 | 0.002464087 | down-regulated |
| OXGR1 | -4.728987095 | 3.403880766 | 2.32E-10 | 6.01E-08 | down-regulated |
| TMPRSS11A | -4.70380445 | -0.570421404 | 9.95E-07 | 5.46E-05 | down-regulated |
| LIN28B | -4.687363824 | -1.652225979 | 0.000293066 | 0.004278857 | down-regulated |
| HOXC10 | -4.658100437 | -0.504959416 | 1.68E-05 | 0.000477732 | down-regulated |
| ALOX12B | -4.609643063 | 1.918104232 | 9.48E-11 | 2.93E-08 | down-regulated |
| ZIC4 | -4.562533435 | -2.958196932 | 8.63E-05 | 0.00166836 | down-regulated |
| SOX14 | -4.545130008 | 2.473788319 | 0.00185202 | 0.016592167 | down-regulated |
| VSTM2B | -4.521946042 | -0.362238543 | 0.002017321 | 0.017606068 | down-regulated |
| FAM83A | -4.519074139 | 2.488728585 | 3.32E-10 | 7.82E-08 | down-regulated |
| MSLNL | -4.457481494 | 1.905317908 | 0.000178812 | 0.002965891 | down-regulated |
| DBX2 | -4.42922833 | 0.003550145 | 1.99E-07 | 1.46E-05 | down-regulated |
| MAGEA9B | -4.417337264 | -3.089062862 | 0.000251157 | 0.003835928 | down-regulated |
| GPR149 | -4.413905297 | -3.65270343 | 0.000536039 | 0.006771415 | down-regulated |
| FAM83C | -4.406680796 | -0.487812727 | 3.09E-05 | 0.000752681 | down-regulated |
| LGI1 | -4.386892955 | -1.960991087 | 7.19E-05 | 0.001450006 | down-regulated |
| ALX1 | -4.375051316 | -2.790641575 | 7.48E-07 | 4.29E-05 | down-regulated |
| C1orf87 | -4.37499686 | -1.85413692 | 0.000296288 | 0.004306536 | down-regulated |
| TUBB2B | -4.301669358 | 3.209646798 | 6.03E-10 | 1.24E-07 | down-regulated |
| HOXC9 | -4.29443531 | 0.880944745 | 1.15E-07 | 9.06E-06 | down-regulated |
| NR0B1 | -4.293756221 | -0.69417748 | 5.11E-05 | 0.001111847 | down-regulated |
| TRPC4 | -4.278224461 | 0.68037447 | 1.51E-08 | 1.84E-06 | down-regulated |
| TAS1R2 | -4.26969355 | -2.393937459 | 0.000370146 | 0.005099404 | down-regulated |
| ITPRID1 | -4.259288477 | -0.859787322 | 1.18E-06 | 6.18E-05 | down-regulated |
| TRIM49B | -4.25835406 | -1.841182311 | 5.82E-07 | 3.47E-05 | down-regulated |
| ATOH1 | -4.236721867 | -3.552163455 | 4.66E-06 | 0.000175684 | down-regulated |
| POPDC3 | -4.224430094 | -0.816615897 | 1.12E-06 | 5.96E-05 | down-regulated |
| MUC16 | -4.221167531 | 2.032785631 | 3.88E-08 | 3.81E-06 | down-regulated |
| KRTAP4-1 | -4.206295378 | -3.084633276 | 1.13E-05 | 0.000355935 | down-regulated |
| IL1A | -4.200693792 | -0.632923515 | 3.85E-08 | 3.81E-06 | down-regulated |
| C11orf53 | -4.199076357 | 1.662176491 | 3.94E-08 | 3.83E-06 | down-regulated |
| SLC5A7 | -4.180360684 | -0.429532576 | 0.000301602 | 0.004367867 | down-regulated |
| NOL4 | -4.157103091 | 0.214473976 | 2.07E-07 | 1.49E-05 | down-regulated |
| RGS21 | -4.128488704 | 0.371137074 | 6.78E-05 | 0.001376717 | down-regulated |
| UGT8 | -4.108391846 | 1.321576214 | 4.93E-09 | 6.84E-07 | down-regulated |
| KIAA1549L | -4.10293 | 3.24691597 | 3.14E-10 | 7.49E-08 | down-regulated |
| RXFP4 | -4.05699929 | -1.480637331 | 2.51E-07 | 1.76E-05 | down-regulated |
| VIL1 | -4.056562698 | -1.302755196 | 1.99E-08 | 2.26E-06 | down-regulated |
| CBLC | -4.053387818 | 1.753375473 | 7.44E-05 | 0.001492053 | down-regulated |
| PRAMEF20 | -4.014705601 | -3.485337606 | 1.97E-06 | 9.12E-05 | down-regulated |
| HOXC11 | -4.000713608 | -2.874960689 | 0.000138927 | 0.002430879 | down-regulated |
| LCE1F | -3.997590981 | -3.2486656 | 0.003723274 | 0.027862295 | down-regulated |
| KRT23 | -3.973240456 | 2.283583246 | 2.36E-07 | 1.67E-05 | down-regulated |
| PADI3 | -3.972225506 | 0.943948881 | 4.70E-05 | 0.00103915 | down-regulated |
| FGF5 | -3.943621575 | 0.14763296 | 2.17E-06 | 9.72E-05 | down-regulated |
| SPATA31E1 | -3.943021385 | -3.140467113 | 7.63E-06 | 0.000256404 | down-regulated |
| ELF5 | -3.930164334 | 1.522112166 | 1.77E-09 | 2.96E-07 | down-regulated |
| FGFBP1 | -3.914949808 | -1.278971686 | 0.000355409 | 0.004927823 | down-regulated |
| HOXB9 | -3.906459945 | -0.716993406 | 2.50E-06 | 0.00010892 | down-regulated |
| CELA3A | -3.896572791 | -2.52330909 | 2.82E-05 | 0.000701833 | down-regulated |
| ABCB11 | -3.892902239 | -1.939333875 | 0.000129481 | 0.002301595 | down-regulated |
| CTCFL | -3.892537928 | -2.184789139 | 2.62E-08 | 2.79E-06 | down-regulated |
| CHGA | -3.848261154 | 1.019134843 | 8.65E-07 | 4.84E-05 | down-regulated |
| CD24 | -3.828638114 | 5.29127506 | 3.58E-09 | 5.30E-07 | down-regulated |
| B3GNT3 | -3.809673362 | 1.190124292 | 1.23E-08 | 1.55E-06 | down-regulated |
| GABRP | -3.778986303 | 1.779320734 | 2.34E-06 | 0.000103149 | down-regulated |
| CALHM3 | -3.770022458 | -0.473623947 | 3.48E-07 | 2.30E-05 | down-regulated |
| PRAMEF19 | -3.765796028 | -2.348220638 | 2.47E-05 | 0.000632898 | down-regulated |
| HOXC13 | -3.753747385 | -0.858424086 | 1.11E-06 | 5.90E-05 | down-regulated |
| ATP6V1G3 | -3.738444474 | -3.337099969 | 2.07E-05 | 0.00055615 | down-regulated |
| OTOGL | -3.737871613 | 1.284485028 | 4.70E-06 | 0.000176699 | down-regulated |
| ADGRG7 | -3.726192745 | -2.06412428 | 7.13E-05 | 0.001440786 | down-regulated |
| CALHM1 | -3.720936958 | 0.157431218 | 6.49E-08 | 5.75E-06 | down-regulated |
| MYEOV | -3.719051566 | 1.357582565 | 3.03E-10 | 7.33E-08 | down-regulated |
| LTF | -3.712976488 | 6.350351905 | 3.87E-06 | 0.000154809 | down-regulated |
| LY6D | -3.702440955 | 3.279851844 | 0.003412116 | 0.026101228 | down-regulated |
| CHAT | -3.67817941 | -1.603615659 | 1.24E-05 | 0.000379602 | down-regulated |
| TRIM49C | -3.67035191 | -3.325286372 | 6.91E-06 | 0.000238153 | down-regulated |
| NDP | -3.666917612 | -1.32201328 | 2.01E-05 | 0.000545992 | down-regulated |
| S100P | -3.659540949 | 1.422497442 | 2.04E-06 | 9.38E-05 | down-regulated |
| OR14A16 | -3.636346679 | -4.086922581 | 3.32E-05 | 0.000790073 | down-regulated |
| DLX1 | -3.631265799 | 0.39101281 | 2.62E-06 | 0.000112269 | down-regulated |
| POU2F3 | -3.625774745 | 3.396085377 | 1.10E-08 | 1.40E-06 | down-regulated |
| TAS1R3 | -3.609168542 | 3.996743762 | 1.68E-10 | 4.70E-08 | down-regulated |
| SLC6A15 | -3.607479391 | -0.035477228 | 6.01E-06 | 0.000213573 | down-regulated |
| AADACL2 | -3.593426487 | -1.716871251 | 3.42E-05 | 0.000806 | down-regulated |
| MAGEA1 | -3.58924133 | 0.085617762 | 0.002552037 | 0.020926003 | down-regulated |
| AC106774.4 | -3.587574415 | -2.592842441 | 1.60E-05 | 0.000462601 | down-regulated |
| ANKRD63 | -3.587157307 | -3.280341323 | 7.64E-06 | 0.000256404 | down-regulated |
| MGAM2 | -3.586497951 | -0.448532169 | 2.23E-05 | 0.000590433 | down-regulated |
| MUC5AC | -3.582570362 | -2.868917811 | 4.82E-05 | 0.001061051 | down-regulated |
| PKHD1 | -3.574997794 | -1.329020064 | 1.18E-05 | 0.000367317 | down-regulated |
| TNNT1 | -3.571952543 | 0.853579988 | 3.59E-07 | 2.35E-05 | down-regulated |
| CCDC33 | -3.539133319 | -2.124391798 | 2.95E-06 | 0.000122371 | down-regulated |
| MUC13 | -3.503219233 | -1.091106879 | 1.32E-06 | 6.70E-05 | down-regulated |
| GABRG2 | -3.498258509 | 0.10334668 | 0.001248842 | 0.012495876 | down-regulated |
| MIA | -3.49635734 | -0.020046996 | 8.09E-08 | 6.83E-06 | down-regulated |
| TRIM43 | -3.48159851 | -0.974833986 | 3.49E-07 | 2.30E-05 | down-regulated |
| LIX1 | -3.477071588 | -1.994569581 | 3.42E-05 | 0.000806 | down-regulated |
| TRIM64B | -3.467950783 | -3.657614957 | 2.34E-05 | 0.00060631 | down-regulated |
| TRIM43B | -3.467212184 | -1.818498023 | 2.89E-07 | 1.98E-05 | down-regulated |
| GNAT3 | -3.449958769 | -0.420025529 | 0.000170534 | 0.002870523 | down-regulated |
| ARMC3 | -3.437030548 | -1.168985534 | 0.000208787 | 0.003316403 | down-regulated |
| TRIML2 | -3.4160952 | -2.381711989 | 0.001054825 | 0.011041737 | down-regulated |
| TMEM151B | -3.416021914 | 1.590569234 | 6.19E-07 | 3.67E-05 | down-regulated |
| KRT4 | -3.414950896 | 0.461778937 | 0.00028448 | 0.004201471 | down-regulated |
| PYDC1 | -3.405467044 | 1.518701272 | 0.004331295 | 0.031101696 | down-regulated |
| CAPNS2 | -3.39611758 | 0.838037219 | 0.001039466 | 0.010925682 | down-regulated |
| ADH1A | -3.386247468 | -0.518078383 | 2.02E-06 | 9.29E-05 | down-regulated |
| PSG3 | -3.382228001 | -2.0949939 | 0.000169632 | 0.002861844 | down-regulated |
| DACH2 | -3.380191939 | -1.427633876 | 2.06E-07 | 1.49E-05 | down-regulated |
| TBC1D29 | -3.376063645 | -0.722752689 | 1.55E-07 | 1.18E-05 | down-regulated |
| OPN5 | -3.370458361 | -3.215075308 | 0.002471857 | 0.020512861 | down-regulated |
| PITX2 | -3.369857217 | -1.460236331 | 3.97E-07 | 2.52E-05 | down-regulated |
| SSX1 | -3.356210339 | -2.517594156 | 0.000364347 | 0.005040034 | down-regulated |
| PIRT | -3.355209724 | -3.579136279 | 9.90E-05 | 0.001856319 | down-regulated |
| TRPM5 | -3.338827433 | 3.006886836 | 4.38E-09 | 6.32E-07 | down-regulated |
| DHRS2 | -3.326201098 | 6.148606381 | 1.75E-05 | 0.000488982 | down-regulated |
| GPR151 | -3.320735284 | -3.253133596 | 5.59E-05 | 0.001190641 | down-regulated |
| KRTAP19-1 | -3.320453253 | -3.839910722 | 0.002248977 | 0.019160727 | down-regulated |
| MOGAT2 | -3.301218038 | -2.174234848 | 0.001205721 | 0.012165921 | down-regulated |
| CEACAM16 | -3.295906828 | -1.046867218 | 0.000392769 | 0.005306088 | down-regulated |
| FOXL2 | -3.273530606 | -0.554030553 | 0.002551241 | 0.020926003 | down-regulated |
| A2ML1 | -3.260759425 | 1.117121985 | 1.24E-06 | 6.42E-05 | down-regulated |
| CACNG6 | -3.251729915 | -3.311213262 | 0.000210173 | 0.003332241 | down-regulated |
| SLCO1B1 | -3.242412477 | -2.803844628 | 0.000106302 | 0.001961624 | down-regulated |
| HAS1 | -3.23603891 | 1.451132354 | 1.17E-05 | 0.000365474 | down-regulated |
| DLX2 | -3.23418989 | -0.109921809 | 9.85E-07 | 5.43E-05 | down-regulated |
| HMX2 | -3.231625943 | -2.905741444 | 0.000861676 | 0.009503386 | down-regulated |
| BRDT | -3.226541685 | -1.139636718 | 0.000947306 | 0.010172031 | down-regulated |
| PITX1 | -3.222244262 | 2.598377537 | 0.000129957 | 0.002305475 | down-regulated |
| MUC4 | -3.199247139 | 3.769919029 | 8.00E-08 | 6.80E-06 | down-regulated |
| HOXC6 | -3.197431291 | 1.738916308 | 4.24E-06 | 0.000166017 | down-regulated |
| HOXC8 | -3.185694585 | 1.22794344 | 0.000110103 | 0.002017172 | down-regulated |
| PRAMEF12 | -3.179260281 | -3.308623603 | 6.16E-06 | 0.00021736 | down-regulated |
| IAPP | -3.17160789 | -2.788665552 | 0.000289219 | 0.004246938 | down-regulated |
| OR5M11 | -3.152635132 | -3.492364948 | 1.70E-05 | 0.000482633 | down-regulated |
| KCNK3 | -3.144696457 | 0.739208568 | 6.98E-05 | 0.001413149 | down-regulated |
| H3.Y | -3.141473211 | 0.267373934 | 2.93E-05 | 0.000722081 | down-regulated |
| PGLYRP3 | -3.136472835 | -2.647346298 | 0.000368767 | 0.005085465 | down-regulated |
| XKR7 | -3.135550842 | -1.411015256 | 8.59E-07 | 4.83E-05 | down-regulated |
| GABRA3 | -3.119274233 | -0.042256737 | 5.08E-07 | 3.09E-05 | down-regulated |
| CD177 | -3.118811714 | 0.547272087 | 3.93E-07 | 2.52E-05 | down-regulated |
| HMX1 | -3.115718229 | -2.435316271 | 0.000284434 | 0.004201471 | down-regulated |
| UGT2B11 | -3.110839819 | -3.806661075 | 0.000348914 | 0.004871727 | down-regulated |
| TRIM51 | -3.105206318 | -3.731078533 | 0.000419107 | 0.005586011 | down-regulated |
| GNG13 | -3.096849076 | 2.955933382 | 1.94E-07 | 1.44E-05 | down-regulated |
| FMN2 | -3.093057497 | -1.467431904 | 8.79E-05 | 0.001691891 | down-regulated |
| ELF3 | -3.092156714 | 4.944567368 | 4.15E-09 | 6.04E-07 | down-regulated |
| FGF19 | -3.083802206 | -3.898486463 | 0.000275677 | 0.004105342 | down-regulated |
| PRAMEF8 | -3.082259782 | -3.66271327 | 4.37E-05 | 0.000982827 | down-regulated |
| SCGB2A1 | -3.082014725 | -1.704391721 | 0.001027534 | 0.010820589 | down-regulated |
| LCN2 | -3.077771476 | 2.665944341 | 1.71E-05 | 0.00048408 | down-regulated |
| PPP1R1B | -3.075043885 | 4.233795932 | 4.51E-06 | 0.000172245 | down-regulated |
| ACTL8 | -3.074041184 | -1.008595763 | 3.66E-05 | 0.000847081 | down-regulated |
| CSF3 | -3.071861727 | 0.479849011 | 0.001666361 | 0.015352543 | down-regulated |
| PRH2 | -3.04901008 | 2.602593002 | 5.61E-06 | 0.00020462 | down-regulated |
| SERPINB13 | -3.042184295 | -2.62603918 | 0.000881892 | 0.009648572 | down-regulated |
| PASD1 | -3.033691226 | -0.841545982 | 0.000705375 | 0.008279485 | down-regulated |
| RNF17 | -3.029455523 | -1.391590158 | 1.82E-05 | 0.000506121 | down-regulated |
| CYP26A1 | -3.016001732 | 1.997232684 | 1.96E-05 | 0.000537539 | down-regulated |
| EN1 | -3.015201408 | -0.405425878 | 0.000658864 | 0.007875583 | down-regulated |
| OTOP1 | -3.007348061 | -1.50318345 | 0.000226365 | 0.003545002 | down-regulated |
| TMPRSS4 | -3.007111772 | 3.282891261 | 8.22E-05 | 0.001606282 | down-regulated |
| PAX2 | -3.004214169 | -2.331381272 | 7.46E-05 | 0.00149422 | down-regulated |
| CELF3 | -2.983962319 | -1.658406897 | 1.01E-06 | 5.54E-05 | down-regulated |
| RIPPLY2 | -2.97699134 | -2.424429757 | 0.007609015 | 0.046250458 | down-regulated |
| KRTAP2-3 | -2.973738433 | -2.709217588 | 0.000969355 | 0.010346722 | down-regulated |
| B4GALNT2 | -2.962971424 | -1.987357145 | 0.000851614 | 0.009438943 | down-regulated |
| SPDEF | -2.96157986 | -1.177370686 | 3.15E-07 | 2.14E-05 | down-regulated |
| NKX6-1 | -2.959184519 | -2.964156358 | 4.08E-05 | 0.000922682 | down-regulated |
| OVCH2 | -2.95826788 | -0.738914806 | 5.78E-05 | 0.001219412 | down-regulated |
| CGA | -2.933427111 | -3.584583012 | 4.74E-05 | 0.00104566 | down-regulated |
| RNF183 | -2.930949618 | -0.154219952 | 1.74E-06 | 8.36E-05 | down-regulated |
| OR2I1P | -2.929138554 | 2.864620899 | 5.13E-06 | 0.00018951 | down-regulated |
| TNFRSF11B | -2.918150418 | 1.410901074 | 4.66E-06 | 0.000175684 | down-regulated |
| ADGRF2 | -2.910147171 | -2.862109299 | 0.000271017 | 0.004059582 | down-regulated |
| SPAG17 | -2.90912938 | -1.074047287 | 2.27E-05 | 0.000595401 | down-regulated |
| CDK5R2 | -2.903376419 | -3.005377373 | 0.000957006 | 0.010245463 | down-regulated |
| PPP1R17 | -2.900875406 | -3.860232411 | 0.000142466 | 0.002473459 | down-regulated |
| SDR9C7 | -2.899689264 | -2.511330211 | 2.63E-05 | 0.000666923 | down-regulated |
| TRIM48 | -2.896981502 | -2.822001797 | 0.000256546 | 0.003885144 | down-regulated |
| KCNIP1 | -2.895968728 | -1.07095588 | 5.41E-05 | 0.001161528 | down-regulated |
| NKX3-2 | -2.895772803 | 1.009687802 | 3.93E-05 | 0.000896525 | down-regulated |
| ZIC1 | -2.882619247 | -2.17207672 | 0.002543229 | 0.020902605 | down-regulated |
| FAM83E | -2.879615358 | 0.920117542 | 1.76E-06 | 8.38E-05 | down-regulated |
| TRIM31 | -2.861693531 | 0.533617941 | 2.90E-08 | 3.07E-06 | down-regulated |
| 11-Mar | -2.85422779 | -1.39514632 | 0.001206255 | 0.012165921 | down-regulated |
| FRRS1 | -2.853839507 | 1.906109727 | 7.07E-08 | 6.21E-06 | down-regulated |
| BSND | -2.850016745 | -1.421653364 | 0.000154843 | 0.002647273 | down-regulated |
| SLCO1B3 | -2.84490868 | -3.635196498 | 0.00133327 | 0.013109569 | down-regulated |
| FABP7 | -2.815272943 | -0.407832383 | 0.004453064 | 0.031778758 | down-regulated |
| CRYM | -2.811281002 | 0.33512771 | 1.96E-07 | 1.44E-05 | down-regulated |
| LGALS14 | -2.806105405 | -1.844182219 | 0.006670621 | 0.042088165 | down-regulated |
| SERPINB3 | -2.788958358 | -2.319679079 | 0.003640216 | 0.027435733 | down-regulated |
| CRISP3 | -2.788399693 | -2.073889928 | 0.001676482 | 0.015406454 | down-regulated |
| GFY | -2.780872974 | -3.070862258 | 0.00045923 | 0.005995779 | down-regulated |
| NOS2 | -2.779239325 | 1.667305851 | 3.06E-06 | 0.000125916 | down-regulated |
| LBP | -2.772628697 | -1.824033899 | 0.000680881 | 0.008046798 | down-regulated |
| MUC2 | -2.764409171 | 0.021090642 | 0.000341199 | 0.004808975 | down-regulated |
| PRTG | -2.756438138 | 0.742788915 | 1.59E-06 | 7.76E-05 | down-regulated |
| KYNU | -2.745969118 | 3.516931715 | 7.53E-08 | 6.52E-06 | down-regulated |
| VGLL1 | -2.738812888 | -0.389927598 | 0.001992742 | 0.017459716 | down-regulated |
| PDCL2 | -2.738525636 | -3.203490174 | 0.002356323 | 0.019774114 | down-regulated |
| OTOS | -2.731706736 | -1.850953985 | 0.001801857 | 0.016289517 | down-regulated |
| TRIM49 | -2.730352885 | -3.4571632 | 0.000460961 | 0.006013992 | down-regulated |
| CST5 | -2.721502197 | -2.485496709 | 0.0003263 | 0.004635531 | down-regulated |
| UNC13A | -2.710243117 | -0.201062094 | 1.89E-06 | 8.80E-05 | down-regulated |
| ARHGAP40 | -2.706110899 | -2.428582498 | 4.17E-05 | 0.00094224 | down-regulated |
| HCAR3 | -2.702360999 | 0.690959977 | 1.30E-06 | 6.65E-05 | down-regulated |
| PTGIR | -2.701945224 | 3.419328343 | 1.10E-06 | 5.87E-05 | down-regulated |
| CYP26C1 | -2.697938165 | -1.568648281 | 2.07E-05 | 0.00055615 | down-regulated |
| LY6L | -2.682756384 | -4.026306693 | 0.001209245 | 0.012187769 | down-regulated |
| DEFB1 | -2.682003902 | 1.331517225 | 3.96E-07 | 2.52E-05 | down-regulated |
| C10orf53 | -2.681626469 | -3.960607801 | 0.000773593 | 0.008842473 | down-regulated |
| FOXP2 | -2.680840095 | 2.164981356 | 0.000124789 | 0.002227048 | down-regulated |
| FGF3 | -2.672263879 | -4.070702292 | 0.002006909 | 0.017523743 | down-regulated |
| ATP6V1B1 | -2.669449792 | 0.956748072 | 2.50E-07 | 1.76E-05 | down-regulated |
| AZGP1 | -2.668588254 | 2.404419769 | 0.000257791 | 0.003899118 | down-regulated |
| KCNJ6 | -2.667378138 | -1.816523409 | 0.000110442 | 0.002017172 | down-regulated |
| NSG2 | -2.665221992 | -2.606139981 | 0.001000989 | 0.010602196 | down-regulated |
| TGM2 | -2.663547562 | 7.762223867 | 3.53E-08 | 3.59E-06 | down-regulated |
| ARHGAP36 | -2.663236945 | -0.891259421 | 3.84E-05 | 0.000881961 | down-regulated |
| MYBPH | -2.660714641 | -1.197475522 | 0.000162272 | 0.002761091 | down-regulated |
| BRS3 | -2.657319057 | -1.929237493 | 0.000180665 | 0.002986059 | down-regulated |
| OVOL3 | -2.65278413 | 0.231867485 | 2.12E-06 | 9.58E-05 | down-regulated |
| CLDN10 | -2.648402112 | 2.408020485 | 2.26E-05 | 0.000595282 | down-regulated |
| GNB3 | -2.63791483 | 2.430522852 | 1.38E-08 | 1.72E-06 | down-regulated |
| SCN5A | -2.636526171 | 0.542177647 | 6.12E-08 | 5.47E-06 | down-regulated |
| PRAMEF7 | -2.631286657 | -3.816281332 | 0.000647082 | 0.007763244 | down-regulated |
| TAS2R30 | -2.614729793 | -2.176136266 | 3.46E-05 | 0.000809956 | down-regulated |
| APOBEC4 | -2.614667693 | -3.610827852 | 0.000846262 | 0.009417147 | down-regulated |
| CPB1 | -2.611252567 | -0.901154835 | 0.000287466 | 0.004230481 | down-regulated |
| PRR35 | -2.60925877 | -3.023552097 | 0.001341952 | 0.013162162 | down-regulated |
| AIRE | -2.605100384 | 2.124696598 | 0.000158138 | 0.00269331 | down-regulated |
| PTH2R | -2.603811761 | -1.335356036 | 5.67E-06 | 0.00020574 | down-regulated |
| CSAG3 | -2.596363483 | -2.549747413 | 0.002231006 | 0.019052961 | down-regulated |
| GSC | -2.593561936 | 1.536483257 | 9.32E-06 | 0.000301564 | down-regulated |
| CDH22 | -2.591506875 | -0.614939182 | 0.000200534 | 0.003231934 | down-regulated |
| CD70 | -2.580493244 | 2.031273191 | 4.97E-06 | 0.000184488 | down-regulated |
| ALOXE3 | -2.576503909 | -0.974664684 | 3.50E-07 | 2.30E-05 | down-regulated |
| PTGS2 | -2.573544209 | 3.454289534 | 4.63E-05 | 0.001027373 | down-regulated |
| ICAM5 | -2.572853322 | 2.357294084 | 3.69E-06 | 0.000148083 | down-regulated |
| ADH1C | -2.572068915 | 1.277323909 | 1.92E-05 | 0.000528185 | down-regulated |
| RNF223 | -2.562333556 | -2.799208659 | 2.94E-06 | 0.000122164 | down-regulated |
| CCL11 | -2.560253582 | -0.627594827 | 1.41E-05 | 0.000418482 | down-regulated |
| SHOX2 | -2.559642734 | -1.491920273 | 0.000175842 | 0.002935216 | down-regulated |
| TAS2R60 | -2.559617522 | -1.638391168 | 0.000410735 | 0.005504879 | down-regulated |
| DNER | -2.55701313 | 1.311903464 | 1.39E-05 | 0.000413615 | down-regulated |
| KCTD8 | -2.553375972 | -1.251197399 | 9.13E-05 | 0.001740996 | down-regulated |
| PDIA2 | -2.551856544 | 0.138121374 | 5.92E-06 | 0.000212009 | down-regulated |
| CLDN4 | -2.54409182 | 5.177263682 | 4.42E-06 | 0.000170249 | down-regulated |
| ANKRD33 | -2.541076783 | -3.714325909 | 0.00029011 | 0.004255338 | down-regulated |
| HTR7 | -2.539156771 | 0.76246439 | 7.75E-07 | 4.43E-05 | down-regulated |
| HOXA6 | -2.536190094 | -1.75005018 | 0.000112839 | 0.002048496 | down-regulated |
| KLHDC7A | -2.535778188 | -1.068645071 | 0.000536665 | 0.006774542 | down-regulated |
| MT3 | -2.527517099 | 2.494676732 | 0.000385758 | 0.005254992 | down-regulated |
| UBD | -2.526204944 | 4.221807166 | 5.31E-05 | 0.001144345 | down-regulated |
| GRIA1 | -2.514567375 | -0.248828557 | 0.004513666 | 0.032010546 | down-regulated |
| UNC5A | -2.509449747 | -0.231612009 | 1.24E-05 | 0.000379602 | down-regulated |
| BHLHA15 | -2.508916254 | -1.719998636 | 0.000306558 | 0.00442166 | down-regulated |
| LEMD1 | -2.507622431 | -0.692951056 | 0.000517141 | 0.006575224 | down-regulated |
| FAM9A | -2.504018449 | -3.604909621 | 0.000285203 | 0.004206233 | down-regulated |
| FNDC1 | -2.503701647 | 2.079428858 | 1.70E-05 | 0.000482633 | down-regulated |
| CYP24A1 | -2.501958715 | 0.971519889 | 7.77E-05 | 0.001532558 | down-regulated |
| SH2D6 | -2.501846062 | 0.798128146 | 1.10E-06 | 5.87E-05 | down-regulated |
| KHDC1L | -2.501364214 | -0.539793263 | 2.90E-06 | 0.000121419 | down-regulated |
| PAX4 | -2.500120049 | -3.599182625 | 0.001336255 | 0.013120657 | down-regulated |
| SOX8 | -2.495590339 | 2.513724463 | 2.27E-05 | 0.000595401 | down-regulated |
| SNCB | -2.480856462 | -1.223331322 | 0.004713235 | 0.032994491 | down-regulated |
| SLC26A9 | -2.480695967 | 0.767529144 | 0.000433078 | 0.005725335 | down-regulated |
| AHSG | -2.475622801 | -3.485329619 | 0.000986026 | 0.010493381 | down-regulated |
| CFHR3 | -2.467616588 | 0.969411053 | 9.38E-06 | 0.000302943 | down-regulated |
| CHRDL2 | -2.461040939 | -1.636932098 | 9.86E-05 | 0.001850758 | down-regulated |
| CLDN3 | -2.459060127 | 3.786536909 | 9.84E-05 | 0.001849116 | down-regulated |
| TAC1 | -2.457379424 | 0.749855172 | 0.005935318 | 0.038732117 | down-regulated |
| SERPINB2 | -2.45569439 | 0.065776236 | 0.000551371 | 0.006916292 | down-regulated |
| POTEF | -2.446744763 | 1.146230589 | 9.23E-05 | 0.001751078 | down-regulated |
| OR1G1 | -2.441985184 | -3.26453934 | 4.55E-06 | 0.000173384 | down-regulated |
| DDN | -2.439552203 | -1.126411852 | 4.87E-06 | 0.00018147 | down-regulated |
| SYNGR3 | -2.433230043 | 1.367183168 | 1.26E-07 | 9.85E-06 | down-regulated |
| SSX3 | -2.431580667 | -3.706202268 | 0.000860865 | 0.009500299 | down-regulated |
| C15orf48 | -2.426560809 | 2.637537923 | 1.18E-06 | 6.18E-05 | down-regulated |
| MGAT3 | -2.419629692 | 4.699092325 | 3.58E-09 | 5.30E-07 | down-regulated |
| TAS2R42 | -2.414860461 | -2.950221966 | 0.000675292 | 0.008002175 | down-regulated |
| CA9 | -2.413929364 | 2.030204024 | 4.83E-05 | 0.001061051 | down-regulated |
| NKX2-5 | -2.413543229 | -3.092931257 | 0.007189301 | 0.044390651 | down-regulated |
| KIF1A | -2.408289895 | 4.904261688 | 0.000101884 | 0.001897728 | down-regulated |
| TAS2R13 | -2.404641717 | -2.082432077 | 0.000765122 | 0.008756832 | down-regulated |
| EPPIN | -2.403730468 | -4.113706786 | 0.002496872 | 0.020662974 | down-regulated |
| SIGLEC15 | -2.403400997 | 3.028004882 | 7.27E-06 | 0.000247564 | down-regulated |
| OTX1 | -2.402231969 | 0.590520301 | 0.000377448 | 0.005185202 | down-regulated |
| HCAR2 | -2.390074708 | 3.464203478 | 1.05E-07 | 8.40E-06 | down-regulated |
| SLC5A4 | -2.385612023 | 1.255016769 | 0.000324071 | 0.00460752 | down-regulated |
| MUC5B | -2.380520721 | 0.026738221 | 0.000203016 | 0.003256267 | down-regulated |
| ERBB3 | -2.379761825 | 3.777219319 | 1.21E-05 | 0.000375193 | down-regulated |
| FOXA1 | -2.377758296 | 1.955188618 | 0.007796023 | 0.046986133 | down-regulated |
| MATN4 | -2.375546638 | 2.186091756 | 0.00036803 | 0.005083133 | down-regulated |
| KRT40 | -2.365966784 | -1.360973233 | 6.51E-06 | 0.000227446 | down-regulated |
| SLC35F1 | -2.35985934 | -0.673090812 | 2.20E-05 | 0.000584806 | down-regulated |
| HTR2C | -2.34804187 | 0.45410485 | 0.004706022 | 0.032969779 | down-regulated |
| GABRG1 | -2.333968746 | -3.430836691 | 0.001317982 | 0.01301093 | down-regulated |
| NRG3 | -2.328341939 | -1.656864704 | 0.00116613 | 0.011907436 | down-regulated |
| CORIN | -2.327592122 | 1.541606578 | 5.12E-07 | 3.10E-05 | down-regulated |
| MAGEA11 | -2.323491224 | -3.590849239 | 0.00115158 | 0.011826325 | down-regulated |
| FOXD1 | -2.321116341 | -0.403455125 | 0.000253553 | 0.003862636 | down-regulated |
| LMX1B | -2.318708177 | 0.73775494 | 0.001485327 | 0.01418749 | down-regulated |
| CELF4 | -2.313772428 | -0.072357669 | 3.92E-06 | 0.000155944 | down-regulated |
| MSX2 | -2.312884867 | -1.370858257 | 0.000185501 | 0.003049091 | down-regulated |
| FAM43B | -2.304011115 | -0.479270135 | 0.000762268 | 0.008745377 | down-regulated |
| RAB27B | -2.302433726 | 2.709924177 | 9.45E-08 | 7.74E-06 | down-regulated |
| CHI3L1 | -2.299057431 | 5.840780223 | 6.15E-05 | 0.001281695 | down-regulated |
| 4-Mar | -2.297925027 | -1.529759188 | 3.70E-05 | 0.00085433 | down-regulated |
| TWIST1 | -2.296832649 | 3.125141914 | 8.52E-06 | 0.000279974 | down-regulated |
| BCL2A1 | -2.296643519 | 3.674680347 | 8.41E-06 | 0.000277284 | down-regulated |
| RASAL1 | -2.29471181 | 2.793578925 | 1.83E-06 | 8.62E-05 | down-regulated |
| CXCL1 | -2.282872897 | 3.355602275 | 9.77E-05 | 0.001837026 | down-regulated |
| LRAT | -2.281414882 | 2.9721091 | 2.15E-06 | 9.64E-05 | down-regulated |
| CELA3B | -2.27678649 | -2.44635819 | 0.000937305 | 0.010082785 | down-regulated |
| TMEM174 | -2.276582424 | -2.721128771 | 0.000470296 | 0.006104643 | down-regulated |
| PIGR | -2.276416321 | 2.191918575 | 0.000708435 | 0.00829907 | down-regulated |
| IL36RN | -2.274543382 | -2.449657006 | 0.001326305 | 0.013067958 | down-regulated |
| ARX | -2.269049891 | -3.014456769 | 0.001396023 | 0.013558773 | down-regulated |
| CXCL13 | -2.267538905 | 4.131655299 | 0.000380946 | 0.005205299 | down-regulated |
| DLX5 | -2.266963919 | 2.348144452 | 0.000949551 | 0.010190031 | down-regulated |
| ASCL4 | -2.265899923 | 1.006366389 | 0.000423668 | 0.005634221 | down-regulated |
| SMYD1 | -2.25981377 | -3.534834522 | 0.007715453 | 0.04665764 | down-regulated |
| ADORA1 | -2.255127312 | 3.014257912 | 1.03E-05 | 0.000326418 | down-regulated |
| ETV4 | -2.253857083 | 2.135907078 | 2.52E-06 | 0.000109185 | down-regulated |
| TBXT | -2.250776481 | -3.901342609 | 0.001477243 | 0.014155599 | down-regulated |
| PLEKHS1 | -2.236251118 | 1.315560924 | 0.001422326 | 0.013739687 | down-regulated |
| GLYATL2 | -2.234600358 | -0.336167406 | 0.000108362 | 0.001993501 | down-regulated |
| ADGRF4 | -2.231295863 | -2.05574148 | 0.003567396 | 0.027000585 | down-regulated |
| CYB5R2 | -2.22774254 | 2.48429395 | 2.55E-08 | 2.73E-06 | down-regulated |
| CTNNA2 | -2.225215535 | -0.459449894 | 0.000294156 | 0.004284288 | down-regulated |
| KCNJ13 | -2.225016942 | -0.529794876 | 0.001800289 | 0.016289517 | down-regulated |
| CREB3L1 | -2.224227715 | 4.49803495 | 9.31E-06 | 0.000301564 | down-regulated |
| FABP6 | -2.205730038 | 1.329150064 | 0.002400287 | 0.020077169 | down-regulated |
| SLC9A2 | -2.204742034 | 1.180186289 | 1.45E-07 | 1.11E-05 | down-regulated |
| IL12A | -2.200880413 | -0.180936406 | 4.94E-06 | 0.000184008 | down-regulated |
| KIAA1211 | -2.199081137 | 0.595912411 | 3.85E-05 | 0.000883455 | down-regulated |
| PTPRQ | -2.195811271 | -2.015184724 | 0.001283669 | 0.012772466 | down-regulated |
| DPEP1 | -2.191862297 | 2.79506202 | 1.79E-05 | 0.000499127 | down-regulated |
| KCNN1 | -2.185783091 | 1.362348272 | 6.82E-07 | 3.99E-05 | down-regulated |
| TACSTD2 | -2.181355193 | 5.876102042 | 2.32E-06 | 0.000102729 | down-regulated |
| SPON1 | -2.18023615 | 2.931203338 | 6.10E-05 | 0.001274612 | down-regulated |
| WDR87 | -2.177003892 | -0.806214299 | 0.000182229 | 0.003009136 | down-regulated |
| ADAMTS8 | -2.176207405 | 1.201213112 | 2.62E-06 | 0.000112269 | down-regulated |
| BICDL2 | -2.175692742 | 2.127573678 | 0.000179463 | 0.00297168 | down-regulated |
| CYP4Z1 | -2.175260633 | -2.139611158 | 7.50E-05 | 0.001499652 | down-regulated |
| ALKAL1 | -2.167560308 | -2.545144191 | 0.0024713 | 0.020512861 | down-regulated |
| PITX3 | -2.164810297 | -3.57530938 | 0.002300462 | 0.019447054 | down-regulated |
| ZNF804A | -2.159653074 | -1.244054077 | 4.93E-05 | 0.001079921 | down-regulated |
| GJB7 | -2.158143039 | 1.440738166 | 0.000206689 | 0.003297442 | down-regulated |
| DTNA | -2.152305215 | 2.542133066 | 2.28E-05 | 0.00059672 | down-regulated |
| PAPPA2 | -2.145401945 | 1.468637896 | 0.002522813 | 0.020791142 | down-regulated |
| GUCA1A | -2.140432863 | -2.295466284 | 0.000637018 | 0.007688894 | down-regulated |
| TRPC6 | -2.134434392 | 1.765171833 | 2.23E-06 | 9.92E-05 | down-regulated |
| WDR72 | -2.134325718 | 5.114977146 | 0.000322905 | 0.004594601 | down-regulated |
| NEUROD1 | -2.129248176 | -3.380917851 | 0.004579445 | 0.032361656 | down-regulated |
| C2orf72 | -2.126919469 | -1.441798163 | 0.000385077 | 0.005251813 | down-regulated |
| TENM2 | -2.122808977 | 5.294810055 | 5.09E-05 | 0.00111118 | down-regulated |
| SSX5 | -2.121168377 | -3.939201123 | 0.00045095 | 0.00591356 | down-regulated |
| KRT80 | -2.111811325 | 1.358660879 | 0.000110448 | 0.002017172 | down-regulated |
| PRSS22 | -2.111672642 | 1.539084795 | 2.06E-05 | 0.000555547 | down-regulated |
| SLCO1A2 | -2.108313048 | 1.966309705 | 0.001170582 | 0.011936123 | down-regulated |
| CHRNB2 | -2.100493138 | 0.312593753 | 0.000304927 | 0.004408874 | down-regulated |
| SPIC | -2.100370697 | 1.967719157 | 0.001794007 | 0.01624316 | down-regulated |
| SPINK1 | -2.09641307 | -2.845574721 | 0.000695323 | 0.008189155 | down-regulated |
| ADAMTS20 | -2.091643612 | 1.902935644 | 0.002929432 | 0.023222688 | down-regulated |
| MN1 | -2.088249856 | 4.370853627 | 2.94E-06 | 0.000122164 | down-regulated |
| TNFSF9 | -2.08690758 | 1.475593016 | 1.27E-05 | 0.00038638 | down-regulated |
| OTP | -2.085295351 | -3.423444751 | 0.005870089 | 0.038505759 | down-regulated |
| GRM7 | -2.081597033 | -1.220282273 | 0.000154372 | 0.002641736 | down-regulated |
| FCRL2 | -2.080874895 | 1.923057137 | 0.001201768 | 0.012153475 | down-regulated |
| WFDC5 | -2.07678298 | -0.415533993 | 0.005732959 | 0.037825275 | down-regulated |
| IQCM | -2.075736461 | -1.769804931 | 0.000417725 | 0.005571744 | down-regulated |
| ITGB8 | -2.069961672 | 3.617021171 | 3.15E-05 | 0.000761426 | down-regulated |
| BECN2 | -2.063956899 | -4.062908472 | 0.000468954 | 0.006092645 | down-regulated |
| FAM163B | -2.059627192 | 0.590507571 | 0.000414603 | 0.005542487 | down-regulated |
| CHODL | -2.055980419 | 1.27903185 | 1.99E-06 | 9.18E-05 | down-regulated |
| ALX3 | -2.050912626 | -2.132866873 | 0.007195095 | 0.044411106 | down-regulated |
| CXCL10 | -2.050911914 | 4.957046294 | 5.12E-06 | 0.000189364 | down-regulated |
| NTS | -2.049354607 | 2.068673957 | 0.002573595 | 0.021006547 | down-regulated |
| IL17REL | -2.047105163 | 2.820580748 | 0.00011055 | 0.002017172 | down-regulated |
| GFRA3 | -2.042432912 | 4.046798187 | 0.001454609 | 0.013976119 | down-regulated |
| GPR158 | -2.039266689 | 2.261302427 | 0.002491316 | 0.020645235 | down-regulated |
| ADAMTS19 | -2.037992094 | -1.696369628 | 0.000725327 | 0.008446791 | down-regulated |
| VTCN1 | -2.032481173 | -2.336371997 | 0.005047351 | 0.034735707 | down-regulated |
| FCRL5 | -2.029840191 | 3.064239808 | 0.000522497 | 0.006628415 | down-regulated |
| SLITRK3 | -2.025961855 | -3.386629917 | 0.006431466 | 0.040954551 | down-regulated |
| AWAT1 | -2.024495997 | -3.878482903 | 0.000200137 | 0.003231934 | down-regulated |
| GAD1 | -2.024012047 | 0.592546609 | 3.45E-05 | 0.000809956 | down-regulated |
| RFPL4B | -2.022168117 | -3.271665216 | 0.000569362 | 0.007081955 | down-regulated |
| PTHLH | -2.020520771 | 0.063479762 | 0.000354396 | 0.004921401 | down-regulated |
| ARFGEF3 | -2.01796537 | 2.28792071 | 0.00025431 | 0.003870872 | down-regulated |
| ANO1 | -2.01005417 | 5.001274521 | 1.26E-05 | 0.000384483 | down-regulated |
| BRINP2 | -2.007429644 | -0.79277762 | 0.002751796 | 0.022098317 | down-regulated |
| ZG16B | -2.00702066 | 2.161442267 | 7.77E-05 | 0.001532558 | down-regulated |
| NUTM2F | -2.006277298 | -2.980561158 | 0.001273987 | 0.012690247 | down-regulated |
| FUT3 | -2.003967685 | -0.290890855 | 0.000311054 | 0.004472182 | down-regulated |

| Supplementary Table 2 Differentially expressed lncRNAs in thymoma with and without MG | | | | | |
| --- | --- | --- | --- | --- | --- |
| lncRNA Name | logFC | logCPM | PValue | FDR | Expression in thymoma with MG |
|
| AC023421.1 | 2.955861374 | 5.637872956 | 3.38E-16 | 1.51E-12 | up-regulated |
| PHEX-AS1 | 3.948841273 | 5.217483751 | 1.04E-13 | 1.84E-10 | up-regulated |
| AC005532.1 | 2.407791325 | 4.908449059 | 1.24E-13 | 1.84E-10 | up-regulated |
| AC008443.3 | 2.548873551 | 5.994676182 | 1.56E-12 | 1.74E-09 | up-regulated |
| CA3-AS1 | 2.129171477 | 6.521986091 | 3.97E-12 | 3.55E-09 | up-regulated |
| LINC01341 | 2.442281038 | 11.72538847 | 4.99E-12 | 3.71E-09 | up-regulated |
| AC010307.4 | 2.528916194 | 4.525174822 | 8.00E-12 | 5.10E-09 | up-regulated |
| NRG1-IT1 | 5.238051005 | 3.528748547 | 9.28E-12 | 5.17E-09 | up-regulated |
| AL392089.1 | 2.435856003 | 4.555658747 | 1.09E-11 | 5.41E-09 | up-regulated |
| F10-AS1 | 2.038711537 | 3.516938891 | 4.13E-11 | 1.85E-08 | up-regulated |
| ENOX1-AS2 | 5.038101948 | 3.247466445 | 5.45E-11 | 2.21E-08 | up-regulated |
| AC004160.1 | 2.060075263 | 6.145004971 | 2.50E-10 | 9.29E-08 | up-regulated |
| AL139393.1 | 3.067434291 | 3.625994459 | 3.20E-10 | 1.10E-07 | up-regulated |
| AC116312.1 | 2.496234549 | 3.969134054 | 2.66E-09 | 7.43E-07 | up-regulated |
| AC055874.1 | 3.475440443 | 7.378544718 | 6.60E-09 | 1.59E-06 | up-regulated |
| FAM3D-AS1 | 2.450290503 | 4.71432714 | 6.76E-09 | 1.59E-06 | up-regulated |
| EFCAB6-AS1 | 2.111995918 | 5.643030179 | 2.69E-08 | 4.45E-06 | up-regulated |
| AC015660.2 | 2.046443433 | 3.345778279 | 2.81E-08 | 4.48E-06 | up-regulated |
| SLC26A4-AS1 | 2.073670248 | 8.50249379 | 4.50E-08 | 6.70E-06 | up-regulated |
| AC025470.2 | 3.365243667 | 4.177385956 | 4.88E-08 | 7.03E-06 | up-regulated |
| AL163195.2 | 4.050420256 | 4.99794755 | 6.20E-08 | 8.39E-06 | up-regulated |
| AC010608.1 | 2.309851761 | 3.553580669 | 9.33E-08 | 1.17E-05 | up-regulated |
| AC090241.2 | 2.140346629 | 3.68316975 | 9.44E-08 | 1.17E-05 | up-regulated |
| AC120498.10 | 3.096964401 | 3.116510138 | 1.20E-07 | 1.37E-05 | up-regulated |
| AC018563.1 | 2.601297906 | 4.330684111 | 1.27E-07 | 1.41E-05 | up-regulated |
| AL451062.1 | 2.501813477 | 3.573079614 | 1.68E-07 | 1.80E-05 | up-regulated |
| AC004870.2 | 3.196514903 | 3.981576408 | 1.74E-07 | 1.80E-05 | up-regulated |
| ATXN8OS | 3.781804733 | 2.808315536 | 2.60E-07 | 2.52E-05 | up-regulated |
| AC008609.1 | 3.098343424 | 5.188973865 | 4.40E-07 | 3.95E-05 | up-regulated |
| AL354863.1 | 4.406470713 | 8.257422242 | 5.27E-07 | 4.52E-05 | up-regulated |
| TRPC7-AS1 | 3.093771026 | 2.947436763 | 6.74E-07 | 5.53E-05 | up-regulated |
| DGCR9 | 2.053639133 | 6.629270204 | 6.93E-07 | 5.53E-05 | up-regulated |
| AL512785.1 | 3.124753132 | 4.152894316 | 7.07E-07 | 5.53E-05 | up-regulated |
| AC025284.1 | 2.14237812 | 5.720214472 | 1.48E-06 | 0.000108413 | up-regulated |
| AL359399.1 | 2.381772714 | 3.07210415 | 1.61E-06 | 0.000114087 | up-regulated |
| SLC6A1-AS1 | 2.36113103 | 5.08050987 | 2.33E-06 | 0.000155039 | up-regulated |
| AF106564.1 | 2.542691268 | 7.243904002 | 2.38E-06 | 0.000156099 | up-regulated |
| PEX5L-AS2 | 2.054094304 | 3.560650679 | 2.80E-06 | 0.000176334 | up-regulated |
| AC093330.1 | 2.001744999 | 5.084455412 | 2.81E-06 | 0.000176334 | up-regulated |
| AL034397.2 | 2.972459733 | 4.486671758 | 3.39E-06 | 0.000204324 | up-regulated |
| AC084024.3 | 2.145146769 | 3.936567098 | 4.63E-06 | 0.000258047 | up-regulated |
| AC007216.1 | 2.029171733 | 3.669252758 | 1.37E-05 | 0.000525953 | up-regulated |
| AC079467.1 | 3.40186293 | 13.10330344 | 1.56E-05 | 0.000579664 | up-regulated |
| AC004160.2 | 2.165213075 | 4.285670008 | 1.84E-05 | 0.000650529 | up-regulated |
| AL021328.1 | 2.758954502 | 6.180796692 | 2.61E-05 | 0.000857937 | up-regulated |
| AC002044.1 | 2.104896815 | 3.088648653 | 3.59E-05 | 0.001046621 | up-regulated |
| AF131216.3 | 2.344830265 | 6.510747522 | 4.88E-05 | 0.001345192 | up-regulated |
| AL160262.1 | 2.186029223 | 3.104007713 | 8.90E-05 | 0.00228385 | up-regulated |
| GPC5-AS1 | 2.671622475 | 3.319083656 | 9.59E-05 | 0.002400347 | up-regulated |
| AC010547.2 | 2.122136548 | 3.968744912 | 0.000174764 | 0.003696554 | up-regulated |
| AC090970.1 | 2.032923692 | 2.934387953 | 0.000197172 | 0.004018176 | up-regulated |
| AL035425.3 | 2.554999873 | 12.93984513 | 0.000207826 | 0.004178059 | up-regulated |
| AL035425.1 | 2.26370864 | 8.991462207 | 0.00022597 | 0.004462401 | up-regulated |
| HOTTIP | 2.92549663 | 3.087251985 | 0.000249449 | 0.004798664 | up-regulated |
| AC008060.4 | 2.932863871 | 2.815937433 | 0.00111035 | 0.014574978 | up-regulated |
| AP000282.1 | 2.224838922 | 3.898135715 | 0.004544107 | 0.038482633 | up-regulated |
| FAM182A | -2.340433907 | 5.321098197 | 2.58E-08 | 4.43E-06 | down-regulated |
| PROX1-AS1 | -5.473100533 | 5.226191623 | 1.02E-07 | 1.22E-05 | down-regulated |
| TRPM2-AS | -2.293735609 | 8.083080707 | 1.91E-07 | 1.94E-05 | down-regulated |
| PCAT7 | -2.96040145 | 7.011074326 | 3.65E-07 | 3.40E-05 | down-regulated |
| PANTR1 | -4.363189646 | 4.958567187 | 4.42E-07 | 3.95E-05 | down-regulated |
| AC025580.1 | -5.291768385 | 5.206039112 | 5.11E-07 | 4.47E-05 | down-regulated |
| AC108676.1 | -2.634787968 | 5.153365272 | 1.41E-06 | 0.000105212 | down-regulated |
| FAM83A-AS1 | -3.266056936 | 4.173794033 | 3.36E-06 | 0.000204324 | down-regulated |
| HOXC13-AS | -3.630767633 | 5.575258534 | 3.74E-06 | 0.000218741 | down-regulated |
| SLCO4A1-AS1 | -3.962515293 | 5.701077061 | 3.77E-06 | 0.000218741 | down-regulated |
| AC078993.1 | -2.874110729 | 4.09747144 | 4.80E-06 | 0.000258309 | down-regulated |
| HOTAIR | -6.229466866 | 4.825763162 | 5.10E-06 | 0.000270624 | down-regulated |
| AC008464.1 | -4.427779826 | 3.908543587 | 5.38E-06 | 0.000277066 | down-regulated |
| AC122685.1 | -4.583817339 | 4.87382848 | 5.59E-06 | 0.000283578 | down-regulated |
| AL137804.1 | -2.516876723 | 3.225023402 | 5.70E-06 | 0.000285995 | down-regulated |
| AC011700.1 | -4.090216284 | 3.858676118 | 6.55E-06 | 0.000324898 | down-regulated |
| FAM222A-AS1 | -4.157505836 | 4.480631442 | 7.19E-06 | 0.000348612 | down-regulated |
| NAALADL2-AS2 | -4.863497438 | 4.597493486 | 7.31E-06 | 0.00035087 | down-regulated |
| AC009646.2 | -4.591560993 | 5.290294931 | 7.45E-06 | 0.000353774 | down-regulated |
| HOXC-AS2 | -3.774436195 | 5.177451348 | 8.64E-06 | 0.000389652 | down-regulated |
| G2E3-AS1 | -2.867181194 | 6.333442671 | 8.96E-06 | 0.000399734 | down-regulated |
| AL691482.3 | -4.331371618 | 5.143161451 | 9.55E-06 | 0.000417745 | down-regulated |
| AL121721.1 | -3.233274779 | 3.824277201 | 9.73E-06 | 0.000417745 | down-regulated |
| AL772337.2 | -4.489362918 | 5.550932379 | 1.06E-05 | 0.00043832 | down-regulated |
| AC124248.1 | -2.012883266 | 5.776603631 | 1.21E-05 | 0.000488217 | down-regulated |
| AC044810.2 | -2.842344651 | 4.792931072 | 1.34E-05 | 0.000519447 | down-regulated |
| AC023886.1 | -5.253853447 | 4.192270119 | 1.60E-05 | 0.00058654 | down-regulated |
| AC104985.1 | -3.627465509 | 2.95075211 | 1.61E-05 | 0.00058654 | down-regulated |
| MAGEA4-AS1 | -5.579584555 | 4.382409084 | 1.90E-05 | 0.000666033 | down-regulated |
| AC025580.2 | -2.620992792 | 4.257692537 | 2.39E-05 | 0.000820808 | down-regulated |
| DLX6-AS1 | -3.389001656 | 7.494280792 | 2.41E-05 | 0.000820835 | down-regulated |
| TSPEAR-AS1 | -2.175044403 | 8.565495913 | 2.48E-05 | 0.00082651 | down-regulated |
| RHOXF1-AS1 | -2.078042477 | 8.625917644 | 2.66E-05 | 0.000867026 | down-regulated |
| AC025809.1 | -2.522056469 | 3.186035396 | 2.97E-05 | 0.000920178 | down-regulated |
| AP001150.1 | -3.073247043 | 2.870153227 | 3.42E-05 | 0.001018275 | down-regulated |
| TMEM132D-AS1 | -6.646550893 | 4.636538823 | 3.49E-05 | 0.001030464 | down-regulated |
| AP000526.1 | -2.008528258 | 3.014052401 | 3.89E-05 | 0.001121168 | down-regulated |
| HOXC-AS1 | -3.468290741 | 5.350939459 | 6.93E-05 | 0.00184169 | down-regulated |
| AL161908.1 | -3.893054538 | 3.465055089 | 8.27E-05 | 0.002134482 | down-regulated |
| AC104237.3 | -2.76166825 | 3.82633223 | 9.05E-05 | 0.002307272 | down-regulated |
| LL22NC03-63E9.3 | -2.04428945 | 3.823393913 | 9.76E-05 | 0.002400347 | down-regulated |
| AL034346.1 | -3.396579441 | 2.831236094 | 9.83E-05 | 0.002400347 | down-regulated |
| LBX1-AS1 | -4.829447813 | 5.500394552 | 0.000108724 | 0.002581032 | down-regulated |
| TSPEAR-AS2 | -2.013378052 | 8.881781165 | 0.000118482 | 0.002768498 | down-regulated |
| AC090673.1 | -2.009297054 | 5.150373777 | 0.000124416 | 0.0028622 | down-regulated |
| CLDN10-AS1 | -3.814642949 | 3.265448784 | 0.000141187 | 0.003192455 | down-regulated |
| AC100823.1 | -2.789794422 | 5.640687573 | 0.000144377 | 0.003237966 | down-regulated |
| AC009264.1 | -2.928457677 | 6.817436849 | 0.00015394 | 0.003367918 | down-regulated |
| AL513304.1 | -3.210569576 | 3.681689505 | 0.000180163 | 0.003774957 | down-regulated |
| AC137894.1 | -2.181484334 | 5.677324864 | 0.000205484 | 0.004149666 | down-regulated |
| AC003986.3 | -2.025852025 | 3.643196726 | 0.000237301 | 0.004624779 | down-regulated |
| LINC00442 | -3.240286684 | 4.468292179 | 0.000261445 | 0.00496523 | down-regulated |
| Z82214.1 | -3.34765041 | 2.791109072 | 0.000285092 | 0.005323706 | down-regulated |
| AC079062.1 | -3.431247877 | 4.05589083 | 0.000340235 | 0.006162251 | down-regulated |
| AC003986.2 | -2.007620158 | 3.67604122 | 0.000453162 | 0.007574758 | down-regulated |
| AC034154.1 | -2.122328641 | 4.27391382 | 0.000486952 | 0.007931627 | down-regulated |
| AC117386.2 | -2.086247172 | 2.807804304 | 0.000544729 | 0.008699298 | down-regulated |
| AC104365.1 | -3.042337304 | 3.140827378 | 0.000667308 | 0.010199305 | down-regulated |
| AP003025.1 | -2.775395944 | 2.799412529 | 0.000750077 | 0.011158649 | down-regulated |
| AC111000.4 | -3.457218334 | 2.905591839 | 0.000754107 | 0.011181331 | down-regulated |
| AC104248.1 | -2.176317368 | 3.33055656 | 0.00079015 | 0.011486781 | down-regulated |
| AC104237.2 | -2.239285755 | 3.732411136 | 0.000802375 | 0.011589 | down-regulated |
| AL160191.1 | -2.442168709 | 2.906695565 | 0.000861529 | 0.012129352 | down-regulated |
| LINC01587 | -2.011010842 | 3.573527131 | 0.000885434 | 0.012349036 | down-regulated |
| AC022762.1 | -2.219385719 | 3.207126397 | 0.000935595 | 0.012860275 | down-regulated |
| AC106882.1 | -2.009902912 | 3.636319113 | 0.000978611 | 0.013307059 | down-regulated |
| AC024592.2 | -2.022266754 | 3.870968262 | 0.000990574 | 0.013396761 | down-regulated |
| AC013640.1 | -3.160517281 | 3.609952928 | 0.001060414 | 0.014114169 | down-regulated |
| AC012123.1 | -2.072013437 | 5.478659272 | 0.001201083 | 0.015228501 | down-regulated |
| DLGAP1-AS5 | -5.027264466 | 7.386921063 | 0.001227724 | 0.015522187 | down-regulated |
| CASC8 | -2.116268906 | 6.005310297 | 0.001257807 | 0.015812933 | down-regulated |
| MGAT3-AS1 | -2.018963651 | 3.57347825 | 0.00130188 | 0.016139692 | down-regulated |
| AC093895.1 | -2.095781249 | 7.304791744 | 0.001318311 | 0.016283267 | down-regulated |
| AC018358.1 | -4.016930593 | 3.042246678 | 0.001517595 | 0.018100247 | down-regulated |
| CASC19 | -2.953486218 | 4.012523332 | 0.001609973 | 0.018711742 | down-regulated |
| AC141930.1 | -3.261209203 | 2.968769055 | 0.001883481 | 0.020583091 | down-regulated |
| AC092335.1 | -2.870962994 | 2.947655912 | 0.001899908 | 0.020583091 | down-regulated |
| AC110813.1 | -2.157369251 | 3.893867818 | 0.001939357 | 0.020856267 | down-regulated |
| EGFLAM-AS4 | -4.460946345 | 3.803811132 | 0.002325723 | 0.023590233 | down-regulated |
| LSAMP-AS1 | -2.021421143 | 3.960416919 | 0.002678629 | 0.026159123 | down-regulated |
| AL121827.1 | -2.903086503 | 4.033229472 | 0.002818513 | 0.026878256 | down-regulated |
| AP002409.1 | -3.002859724 | 3.028416597 | 0.002967461 | 0.027959545 | down-regulated |
| AC022509.1 | -2.379085229 | 6.216784673 | 0.004765158 | 0.039900373 | down-regulated |
| MEIS1-AS3 | -2.103250304 | 4.788804055 | 0.005724034 | 0.045241192 | down-regulated |
